# The Role of CD200–CD200 Receptor in Human Blood and Lymphatic Endothelial Cells in the Regulation of Skin Tissue Inflammation

**DOI:** 10.3390/cells11061055

**Published:** 2022-03-21

**Authors:** Dominic Rütsche, Katarzyna Michalak-Micka, Dominika Zielinska, Hannah Moll, Ueli Moehrlen, Thomas Biedermann, Agnes S. Klar

**Affiliations:** 1 Tissue Biology Research Unit, Department of Surgery, University Children’s Hospital Zurich, 8032 Zurich, Switzerland; dominic.ruetsche@kispi.uzh.ch (D.R.); katarzyna.micka@kispi.uzh.ch (K.M.-M.); dominika.zielinska@kispi.uzh.ch (D.Z.); hannah.moll@gmx.ch (H.M.); ueli.moehrlen@kispi.uzh.ch (U.M.); thomas.biedermann@kispi.uzh.ch (T.B.); 2 Children’s Research Center, University Children’s Hospital Zurich, 8032 Zurich, Switzerland; 3 Faculty of Medicine, University of Zurich, 8032 Zurich, Switzerland; 4 Department of Pediatric Surgery, University Children’s Hospital Zurich, 8032 Zurich, Switzerland

**Keywords:** CD200 (OX2), CD200 receptor, regenerative medicine, blood capillaries, lymphatic capillaries, angiogenesis–immune cells/myeloid cells, microvascular endothelial cells, skin bioengineering

## Abstract

CD200 is a cell membrane glycoprotein that interacts with its structurally related receptor (CD200R) expressed on immune cells. We characterized CD200–CD200R interactions in human adult/juvenile (j/a) and fetal (f) skin and in in vivo prevascularized skin substitutes (vascDESS) prepared by co-culturing human dermal microvascular endothelial cells (HDMEC), containing both blood (BEC) and lymphatic (LEC) EC. We detected the highest expression of CD200 on lymphatic capillaries in j/a and f skin as well as in vascDESS in vivo, whereas it was only weakly expressed on blood capillaries. Notably, the highest CD200 levels were detected on LEC with enhanced Podoplanin expression, while reduced expression was observed on Podoplanin-low LEC. Further, qRT-PCR analysis revealed upregulated expression of some chemokines, including CC-chemokine ligand 21 (CCL21) in j/aCD200^+^ LEC, as compared to j/aCD200^−^ LEC. The expression of CD200R was mainly detected on myeloid cells such as granulocytes, monocytes/macrophages, T cells in human peripheral blood, and human and rat skin. Functional immunoassays demonstrated specific binding of skin-derived CD200^+^ HDMEC to myeloid CD200R^+^ cells in vitro. Importantly, we confirmed enhanced CD200–CD200R interaction in vascDESS in vivo. We concluded that the CD200–CD200R axis plays a crucial role in regulating tissue inflammation during skin wound healing.

## 1. Introduction

Loss of skin due to congenital diseases or traumatic tissue defects (e.g., burns) result in concomitant loss of its barrier function. Although some superficial skin defects with intact dermal elements may heal without autografting, deep partial-thickness and full-thickness burn wounds require autografting of healthy skin from an uninjured site (the donor site) and its placement on the primary wound site [1]. Although such split-thickness autografts are the standard of care, for example, for large burns, the autograft donor site often results in a secondary wound. Moreover, these sites, but especially the primary wounds, exhibit significant scarring, contracture, and loss of function [1].

Bioengineered human dermo-epidermal full-thickness skin substitute (DESS) [2,3,4,5,6,7,8] offer an alternative to those autografts, showing improved graft take and reduced contraction, shrinkage, and scarring [9,10]. Recently, our group developed a complex human prevascularized DESS (vascDESS) containing a mature blood and lymphatic vascular network that becomes rapidly perfused after transplantation by inosculation [11]. Organotypic capillary networks were self-assembled in vitro by co-culture of human primary dermal microvascular endothelial cells (HDMEC) and human dermal fibroblasts (HDF) in three-dimensional (3D) collagen type I hydrogels [11].

In this study, we were particularly interested in CD200 (OX-2) expression in HDMEC lining blood and lymphatic capillaries of vascDESS in vitro and in vivo. CD200 has a wide but specific tissue distribution that includes certain vascular endothelia, kidney glomeruli, placental cells, and neurons [11,12]. Its cognate receptor CD200R is primarily expressed on myeloid cells and certain subsets of B and T cells [13,14]. Interestingly, CD200 glycoprotein is involved in the regulation of macrophage function and the control of alloimmune and autoimmune responses through its receptor, CD200R. 

The presence of CD200 molecules on the cell surface is essential to interact with CD200R to keep tissue homeostasis and avoid exaggerated immune responses leading to tissue damage [15,16]. Accordingly, CD200-knockout animals demonstrated chronic inflammation of the central nervous system (CNS), the onset of experimental autoimmune encephalomyelitis, and showed higher susceptibility to autoimmune reactions due to impaired CD200–CD200R interaction in response to injury [15,16]. Outside the CNS, the expression of CD200 by hair follicle epithelium in the skin was found to attenuate immune responses in an alopecia model [17]. 

Furthermore, the immunosuppressive capacity of CD200 was demonstrated to regulate also tissue homeostasis in the central and peripheral nervous system [18], vascular endothelia [19], skin [20], and lymphoid cells [21]. Previous studies demonstrated, that during homeostasis, CD200 maintains an adequate level of activated CD200R^+^ macrophages to preserve tissue integrity, whereas reduced expression of CD200 attenuates macrophage-mediated tissue damage [20,22]. Hence, mice lacking CD200 showed a phenotype with increased numbers of activated CD11b^+^ macrophages and granulocytes [15,22]. CD200–CD200R signaling inhibits cytokine release, mast cell degranulation, and cellular infiltration together with increased attraction or expansion of Treg subsets and diminution of T cell effector responses [23]. Further, there are several lines of evidence suggesting that the immunosuppressive capacity of CD200 may also stimulate cancer growth by decreasing the immune response against cancer cells. For example, CD200 was found upregulated in vascular endothelium surrounding cutaneous squamous cell carcinoma (SCC) [24], in metastatic SCC [25], in chronic lymphocytic leukemia [26], and acute myeloid leukemia [27], melanoma [28], and multiple myeloma [29].

The CD200–CD200R link was described to play a crucial role in regulating the immune response, also during skin wound healing [20]. As such, the CD200–CD200R link is crucial for immunomodulation and attenuated immune cell response and, thus, can reduce scar formation in cutaneous wounds [19]. We aimed here to compare BEC/LEC from adult versus fetal skin, as fetal wounds of the first-trimester pregnancy can heal spontaneously without any scarring [30,31]. This is due to a very low immune cell response and reduced inflammation in those wounds [32]. We believe that the CD200–CD200R link, in particular in EC, might play an important role in this regulation.

Here, we report for the first time the characterization of human CD200 and its cognate receptor in human juvenile/adult and fetal skin as well as in bioengineered vascDESS. We demonstrate that CD200 is expressed on both blood and lymphatic capillaries both in vitro and in vivo, whereas CD200R is detected on peripheral blood-derived monocytes, granulocytes, NK, and T-cells. Moreover, we provide functional assays confirming the importance of CD200–CD200R interaction for immune responses and for skin wound healing.

## 2. Materials and Methods

### 2.1. Cell Isolation and Culture

All experiments were performed according to the Declaration of Helsinki Principles and after permission from the Ethics Commission of the Canton Zurich. Human skin-derived dermal microvascular endothelial cells (HDMECs), dermal fibroblasts (HDF), and keratinocytes (KC) were isolated and expanded from fetal skin obtained from Spina Bifida operations between 24 and 26 weeks of gestational age (University Children’s Hospital Zurich, ethics approval: BASEC No. PB_2020-00066), juvenile/adult skin including foreskin, scalp, skin from the hands, abdomen, legs, and arms from children ≤18 years (University Children’s Hospital Zurich, BASEC No. 2018-00269); adult skin donors (18–65 years old) from various body areas such as breast and abdomen (Kantonsspital Aarau, ethics approval: BASEC No. 2018-00269) as previously described [11,33]. HUVEC were purchased from ScienceCell (ordering no 8000, Switzerland) and cultured as previously described [11]. Blood-derived immune cells were collected from buffy coats. Briefly, peripheral blood mononuclear cells (PBMCs) were isolated by Ficoll gradient from blood donated by healthy volunteers. Informed written consent was obtained from all blood donors at the Zurich Blood Donation Center (Zurich Blood Transfusion Service of the Swiss Red Cross, Schlieren, Switzerland; www.zhbsd.ch (accessed on 18 March 2022)) according to the guidelines of the local ethics committee.

### 2.2. Flow Cytometry and Immunohistochemistry

Flow cytometry: The phenotype of freshly isolated and cultured HDMEC and/or BEC and LECs and immune cells was determined by flow cytometry analysis. Cells (5 × 10^5^) were incubated for 30 min at 4 °C in with primary antibodies and live/dead Zombie Aqua (Table 1). Parallel stainings using isotype-matched control antibodies were conducted (Table 1):

Following the incubation with antibodies, cells were washed twice with FACS buffer (0.5% human serum albumin, 0.5 mM EDTA in PBS) and then analyzed by flow cytometry on a FACS ARIA III 4L (BD Biosciences, Allschwil, Switzerland).

Immunohistochemistry: Immunofluorescence staining on cryosections was performed as described before [6,8] (Table 2).

For double immunofluorescence, some of the primary antibodies were pre-labeled with Alexa 488, 647, or 555-conjugated polyclonal goat F(ab′)2 fragments, according to the manufacturer’s instructions (Zenon Mouse IgG Labeling Kit, Molecular Probes, Invitrogen).

### 2.3. Proliferation Assay

The proliferation ability of cultured HDMEC was determined using colorimetric Cell Counting Kit-8 kit (CKK-8) according to manufacturer’s instructions (Cell Counting Kit-8, Sigma, Buchs, Switzerland). Briefly, BECs, CD200^+^ LECs, or CD200^−^ LECs (passage 1–2), isolated and sorted from fetal and juvenile/adult skin samples, were seeded in 24-well plates at the density of 20,000 cells/well. Triplicates were prepared for each cell type. Subsequently, cells were treated with diluted CKK-8 solution for 1 h at 37 °C, and then, the absorbance was measured at a wavelength of 450 nm with a microplate reader (BioTek, Winooski, VT, USA). The procedure was repeated every second day for 11 days. OD values reflect the number of viable cells. Mean absorbance values and SDs were calculated at each time point. 

### 2.4. Cell Viability Assay

For the viability assay on j/aHDMECs (mixtures of BEC:LEC), cells were concomitantly stained with FdA (80 μg/mL; Sigma F7378, Buchs, Switzerland), PI (200 μg/mL; Sigma P4864, Buchs, Switzerland), and Hoechst 33342 (1 μg/mL; Sigma B2261, Buchs, Switzerland) was conducted. For Ki67/CD31 stainings, non-treated cells were fixed with acetone/methanol (1:1) at −20 °C for 5 min and subsequently stained as described above (section “Immunohistochemistry”). Both the viability assay as well as Ki67/CD31 staining were performed every second day for 11 days.

### 2.5. Immunoassay

Sorted CD200^+/−^ BEC and LEC were seeded onto 24 well plates and cultured until confluency. Once confluency was achieved, peripheral blood mononuclear cells (PBMCs) were isolated from fresh whole human blood. For this, buffy coats (Zurich Blood Transfusion Service of the Swiss Red Cross, Schlieren, Switzerland) were diluted with PBS (1:1) and gently layered over an equal volume of Ficoll-Paque PLUS (GE Healthcare, Opfikon, Switzerland) and then centrifuged for 30 min at 400 g without brake. After centrifugation, the PBMC fraction was removed, transferred into a new Falcon tube, and subsequently washed twice with PBS. Following the isolation, PBMCs were immediately sorted (FACS ARIA III 4L (BD Biosciences, Wokingham, UK) into the different lymphocyte subtypes that have been shown to express the CD200 Receptor (CD200R). Next, adherent endothelial cells were stained with CellTracker Deep Red Dye (Invitrogen, Zug, Switzerland), and sorted lymphocytes were stained with CellTracker Red CMTPX Dye (Invitrogen, Switzerland) according to the manufacturer’s instructions. Stained cells were then co-cultured in an incubator at 37 °C for 30 min. The lymphocytes were then added on top of the endothelial cells and left for another 30 min at 37 °C. In the last step, all cells were stained with Hoechst 33342 (Sigma-Aldrich, Buchs, Switzerland), fixed in PFA (4%), and analyzed on the confocal microscope (Leica SP8 inverse CLSM). The quantification was performed using Fiji (ver. 1.53i, NIH, Bethesda, MA, USA) by counting the number of immune cells to endothelial cells (Immune cells/EC ratio).

### 2.6. Clonogenic Assay

To assess the potential of CD200^−^ and CD200^+^ LEC to form colonies in vitro, we performed a clonogenic assay. Sorted CD200^−^ and CD200^+^ LEC (200, 600, and 1000 respectively) were seeded into gelatin-coated 6-wells in EGM-2MV medium (Lonza, Basel, Switzerland). After seeding, the cells were left to proliferate for 14 days with medium change every 2 days. On day 14, the medium was aspirated and the cells were washed once with DPBS (Invitrogen, Zug, Switzerland). Next, 3 mL of a mixture of 6% glutaraldehyde and 0.5% crystal violet solution (each diluted in water, all Sigma-Aldrich, Buchs, Switzerland) was added to each well for 30 min and removed carefully. Cells were washed with water and left to dry. Cell colonies were counted by eye. 

### 2.7. qRT-PCR

Total RNA was isolated from cultured cells either grown on culture dishes or directly following FACS sorting according to the manufacturer’s protocol (RNeasy, Qiagen, Hombrechtikon, Switzerland), including a DNAse treatment to remove genomic DNA. Three biological replicates were collected per condition. RNA purity was assessed using an Epoch spectrophotometer (Take3 micro-volume plate, BioTek, Lucerne, Switzerland). Only pure RNA with absorption ratios A_260_/A_280_ ~2.0–2.1 and A_260_/A_230_ ~2.1–2.3 were used for qRT-PCR. RNA was stored at −80 °C until further use. qRT-PCR was performed according to published protocols [34,35,36,37]. First, single-stranded RNA was converted to cDNA using the GoScript Reverse Transcriptase kit (Promega, Madison, WI, USA), following the manufacturer’s instructions. qRT-PCR was carried out in technical triplicates (*n* = 3) using SYBR green chemistry (PowerTrack SYBR Green Master Mix, Invitrogen, Zug, Switzerland). Each 10 μL reaction contained 5 ng of cDNA, and amplification steps were carried out on a QuantStudio 7 Pro Real-Time PCR System (Applied Biosystems, Invitrogen, Zug, Switzerland). All data were normalized to GAPDH and quantification was performed using the 2^−∆∆CT^ method with efficiency correction (Pfaffl method) [23]. All primers were designed by ourselves except for TGF-B1 [38] and CD200 [39] (Table 3).

### 2.8. Preparation of vascDESS and Non-vascDESS

Collagen type I hydrogels were prepared as previously described [2]. In total 1 × 10^5^ of cells (EC and fibroblasts 1:1) were resuspended in 1 mL of collagen gel. The gels were placed in 6-well cell culture inserts with membranes of 3.0 μm pore-size (BD Falcon, Kaiserslauten, Germany) and kept for 30 min at 37 °C in a humidified incubator containing 5% CO_2_. After the polymerization period, EGM-2MV (Lonza, Basel, Switzerland) was added to the upper and lower chambers of the well/insert and hydrogels were incubated for two weeks. Then, hydrogels were covered by keratinocytes (7.5 × 10^4^/gel), cultured for an additional week, and transplanted onto immuno-incompetent rats [2].

### 2.9. Transplantation of Tissue-Engineered Skin Substitutes

The surgical protocol was approved by the local Committee for Experimental Animal Research (Cantonal veterinary office Zurich, permission number ZH045/2019). Immuno-incompetent female nu/nu rats, eight to ten weeks old (Envigo, Horst, The Netherlands), were anesthetized by inhalation of 5% Isoflurane (Baxter, Volketswil, Switzerland) and maintained by inhalation of 2.5% Isoflurane via mask. The dermo-epidermal skin substitutes were transplanted on full-thickness skin wounds created on the back of the rats. 

vascDESS and non-vascDESS (three independent donors for HDMEC each, *n* = 2 rats per condition (*n* = 6 per condition: 1 and 3 weeks; in total 12 vascDESS and 12 non-vascDESS) were transplanted onto full-thickness skin defects prepared on the backs of the rats. To prevent wound closure from the surrounding rat skin, custom-made steel rings (diameter 2.6 cm) were sutured into full-thickness skin defects using non-absorbable polyester sutures (Ethibond^®^, Ethicon, Raritan, NJ, USA). The transplants were then covered with a silicone foil (Silon-SES, BMS, New York, NY, USA), a polyurethane sponge (Ligasano, Ligamed, Innsbruck, Austria), a cohesive conforming bandage (Sincohaft, Theo Frey AG, Bern, Switzerland), and tape as a wound dressing. Dressing changes and photographic documentation were performed once per week. Animals were euthanized using carbon dioxide and the transplanted skins analogs were harvested after 7 and 21 days by in toto excision and processed for immunohistochemical analysis.

### 2.10. Quantification of CD200 and CD200R on Blood and Lymphatic Capillaries In Vivo

Capillary profiles of immunofluorescently stained normal j/a and f human skin, non-vasc/vascDESS analogs, and rat tissue (lymph nodes) were quantified on 6–8 μm thick cryo-sections using Fiji image analysis software (ver. 1.53i, NIH). The entire view field regions at 10· magnification were counted (*n* = 12 vascDESS and *n* = 12 non-vascDESS in vivo biopsies). First, blood (CD31^+^Podo^−^) and lymphatic (CD31+Podo+) capillaries were quantified in normal j/a and f human skin. Then, the CD200 expression was quantified on those blood and lymphatic capillaries in normal j/a and f human skin.

For some experiments, CD31^+^CollIV^+^ or CD31^+^Podo^+^ were identified as blood or lymphatic capillaries, respectively. Further, CD200 expression was assessed on those blood CD31^+^CollIV^+^ or lymphatic CD31^+^Podo^+^ capillaries in human skin and vascDESS. Furthermore, human CD200R expression was assessed in human skin and in the human CD90-positive dermis of non-vasc/vascDESS analogs. 

### 2.11. Statistical Analysis

All results are reported as mean ± SD. Statistical analysis was performed with GraphPad Prism 4.0 (Graph Pad software, La Jolla, CA, USA). Comparison between two groups was performed using the two-tailed unpaired Student’s *t*-test and between multiple groups using two-way ANOVA with Bonferroni multiple comparisons test.

* indicates *p*-value 0.01 to 0.05 (significant) 

** indicates *p*-value 0.001 to 0.01 (very significant)

*** indicates *p* < 0.001 (extremely significant)

**** indicates *p* < 0.0001 (extremely significant)

ns = not significant (*p* > 0.05)

## 3. Results

### 3.1. CD200 Is Expressed in Normal Human Juvenile/Adult and Fetal Skin

**Figure 1:**
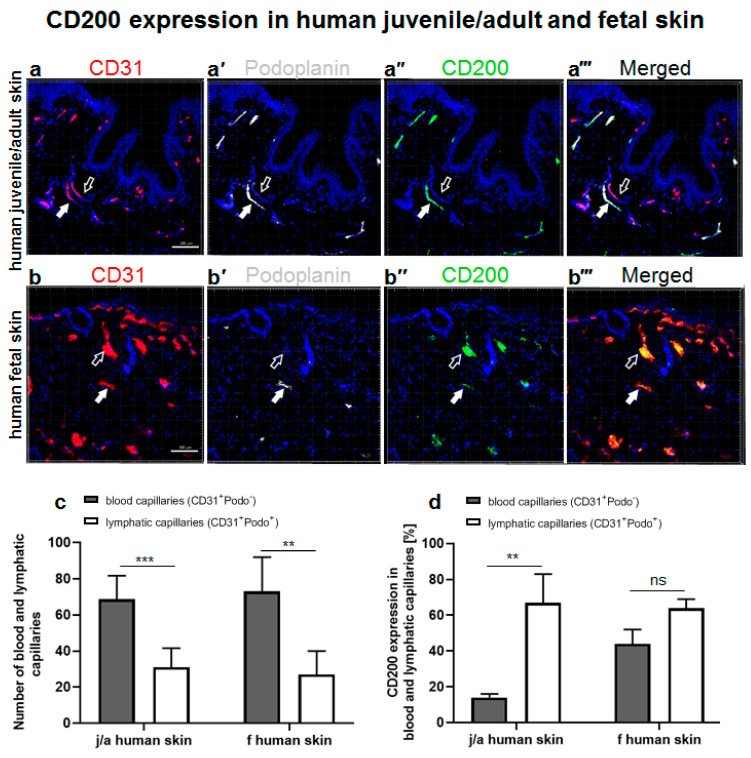
Evaluation of CD200 expression in normal human juvenile/adult and fetal skin. (**a**–**a’’’**) Immunofluorescence triple staining of juvenile/adult (j/a) human skin with antibodies against CD31 (**a**) for human blood capillaries (red), podoplanin (**a’**) depicting human lymphatic endothelium (white), and CD200 (**a’’**) (green). (**a’’’**) represents a merged confocal Z stack. Whereas blood capillaries stain only CD31^+^ (empty arrows), lymphatic capillaries are CD31^+^Podo^+^ (white arrows). Note the presence of blood capillaries (CD31^+^Podo^−^) which are mainly negative for CD200 (empty arrows), as well as the presence of lymphatic capillaries (CD31^+^Podo^+^) showing the expression of CD200 marker (white filled arrows). (**b**–**b’’’**) Immunofluorescence triple staining of human fetal skin (f skin) against CD31 (**b**) delineating human blood capillaries (red), podoplanin (**b’**) depicting human lymphatic capillaries (white), and CD200 (**b’’**) (green). Note the presence of blood and lymphatic capillaries expressing CD200 (empty and filled white arrows, respectively). (**b’’’**) represents a merged overlay. (**c**) Quantification of blood and lymphatic capillaries in j/a and f skin. In the dermis of j/a, blood capillaries constitute approximately 68.8 ± 13%, whereas lymphatic endothelium represents 31.0 ± 10.6% of all vessels present in this skin type (*** *p* < 0.0001). In the f skin, blood endothelium comprises 73.0 ± 19% while lymphatic vessels constitute 27.0 ± 13% of all capillaries (** *p* < 0.001). (**d**) Quantification of CD200 expression on blood and lymphatic capillaries of j/a or f skin. Note the significantly higher expression of CD200 on j/a lymphatic capillaries as compared to the j/a blood capillaries (67.0 ± 16% vs. 14.0 ± 2.0%, respectively, *** *p* < 0.0001), whereas the expression of CD200 was similar in blood and lymphatic capillaries of human f skin (44.0 ± 8.0% vs. 64.0 ± 5.0%, respectively, *p* = ns), *n* = 5 independent j/a and fetal skin donors each. Cell nuclei are stained with Hoechst (blue). Scale bars 100 μm.

The distribution of CD200 ligand on capillaries was examined by immunofluorescence in normal human juvenile/adult (j/a) skin as well as in fetal (f) skin (Figure 1). To distinguish between human blood and lymphatic capillaries, we stained them specifically with human CD31 (red, Figure 1a,b) and podoplanin (white, Figure 1a’,b’) antibody, respectively.

The stainings of podoplanin/CD31-positive lymphatic capillaries in human j/a and f skin were further confirmed by immunofluorescence analysis for PROX1, which is a nuclear lymphatic lineage marker (Supplementary Figure S1a–d). Therefore, podoplanin and PROX1 were used interchangeable as lymphatic markers for various analyses in this study.

Whereas blood capillaries of both j/a and f skin samples were only CD31-positive (empty arrows), lymphatic capillaries showed the expression of podoplanin, a lymphatic endothelium-specific marker, and a faint CD31 co-expression (white arrows). The overall quantification of capillaries located in the dermis of both skin types revealed a significantly higher number of blood capillaries (CD31^+^Podo^−^) as compared to the number of lymphatic capillaries (CD31^+^Podo^+^) (Figure 1c). Specifically, in the j/a skin, blood capillaries constituted 68.8 ± 13.0%, while lymphatic capillaries comprised 31.0 ± 10.6% of all capillaries presented in this skin type (*p* < 0.0001). In fetal skin, blood capillaries represented 73.0 ± 19.0%, whereas lymphatic capillaries counted for 27.0 ± 13.0% of all capillaries (*p* < 0.001).

Further, we detected CD200 on CD31^+^Podo^−^ blood capillaries and CD31^+^Podo^+^ lymphatic capillaries in j/a (Figure 1a’’’) as well as in f skin (Figure 1b’’’). Quantification revealed an enhanced expression of CD200 on lymphatic (68 ± 73%) as compared to blood capillaries (16 ± 97%) in the dermis of j/a skin (*p* = 0.0004) (Figure 1d). By contrast, the differences between the CD200 expression on lymphatics (64 ± 5%) and blood capillaries (44 ± 8%) of fetal skin were not statistically significant (*p* = ns).

### 3.2. CD200 Is Differently Expressed on j/a and Fetal HDMEC Populations In Vitro

**Figure 2:**
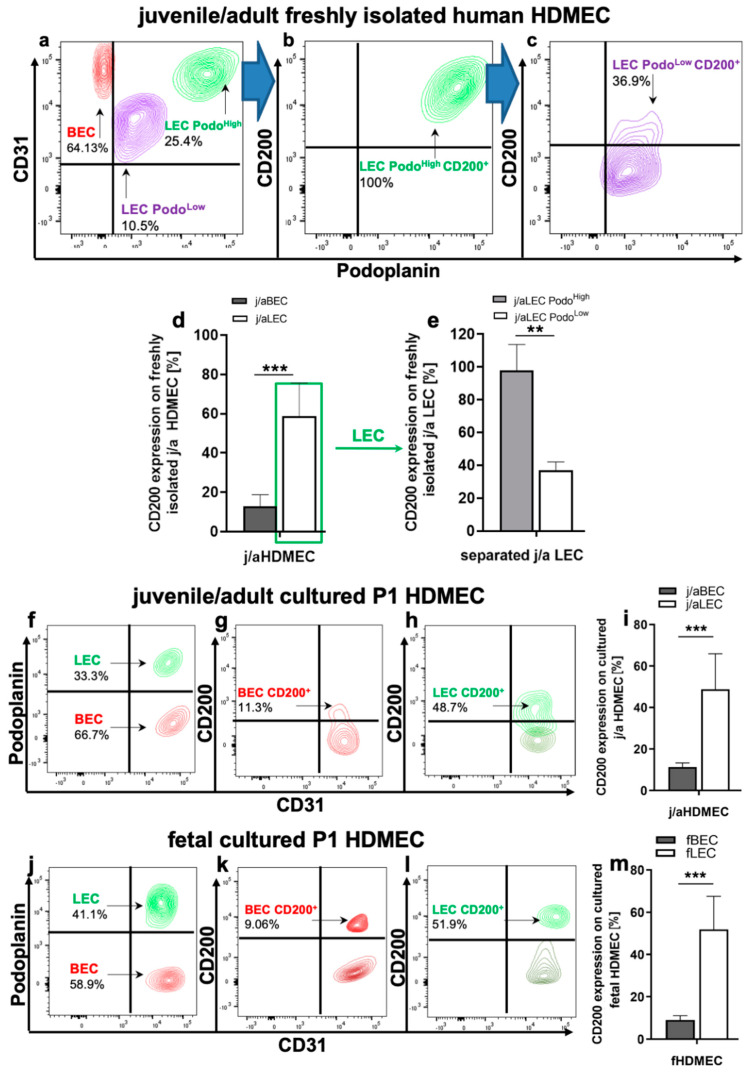
Expression of CD200 on freshly isolated as well as in vitro cultured juvenile/adult and fetal HDMEC. (**a**) Flow cytometry analysis of freshly isolated HDMEC derived from j/a human skin. CD31 and podoplanin were used to discriminate between blood endothelial cells (BEC, CD31^+^Podo^−^) and lymphatic endothelial cells (LEC, CD31^+^Podo^+^). HDMEC obtained from freshly isolated j/a contained 60.13 ± 17.35% of j/aBEC. Note the presence of two distinct subpopulations of LEC expressing different levels of podoplanin, namely LEC Podo^High^, which comprise 26.8 ± 6.75%, as well as LEC Podo^Low^ representing 11.5 ± 2.95% of all freshly isolated j/aHDMEC. (**d**,**e**) Flow cytometric analysis of CD200 expression in freshly isolated j/aBEC and Podo^High^ and Podo^Low^ LEC. Whereas only 12.91 ± 5.86% of freshly harvested j/aBEC stained positive for CD200, 58.73 ± 16.97% of all j/aLEC expressed this marker. Please note that LEC Podo^High^ are almost entirely positive for CD200 (98.1 ± 15.6%) (**b**,**e**), while LEC Podo^Low^ exhibit only a moderate expression of this marker (36.9 ± 5.1%) (*p* = 0.0041) (**c**,**e**). (**f**–**i**) Flow cytometric analysis of cultured HDMEC at passage 1 (P1) derived from j/a skin. Cultured j/aHDMEC contains approximately 67.5 ± 18.21% of BEC as well as 31.8 ± 10.35% of LEC (**f**). Whereas 11.3 ± 2.0% of cultured j/aBEC exhibit CD200 expression (**g**,**i**), a significantly higher number of cultured j/aLEC was CD200-positive (48.7 ± 17.2, *p* < 0.0001) (**h**,**i**). (**j**–**m**) Flow cytometric analysis of cultured fHDMEC at (P1) consisting of 58.4 ± 17.4% of fBEC and 41.6 ± 11.5 of fLEC. Further, 9.06 ± 2.0% of fBEC show the expression of CD200 (k,m), while 51.9 ± 15.7% of fLEC are positive for CD200 (l,m) (*p* < 0.0001). Two-way unpaired student *t*-test with ns = not significant (*p* > 0.05); ** for *p*-value 0.001 to 0.01 (very significant); and *** for *p* < 0.001 (extremely significant) was used for statistical analysis, *n* = 5 independent j/a and fetal skin donors each.

Freshly isolated and in vitro cultured (P1) HDMEC derived from j/a and f skin were analyzed with regard to CD200 expression by flow cytometry (Figure 2). Human CD31 and podoplanin antibodies were used to identify human blood and lymphatic microvascular endothelial cells. Further, the detailed gating strategy is described in Supplementary Figure S2. 

Flow cytometry analysis revealed the presence of CD31^+^Podo^−^ blood endothelial cells (BEC) in the freshly isolated j/aHDMEC, which constitute 64.13 ± 11% of all HDMEC (Figure 2a). Further, we detected altogether 35.9 ± 8% CD31^+^Podo^+^ lymphatic endothelial cells (LEC) in the freshly isolated HDMEC fraction (Figure 2a).

The expression of CD200 on freshly isolated j/aBEC was relatively low and accounted for 12.9 ± 5.9%, whereas CD200 was detected on 58.73 ± 17% of all j/aLEC (*p* = 0.0004) (Figure 2d).

Moreover, we observed the presence of two distinct subpopulations of CD31^+^Podo^+^ lymphatic endothelial cells (LEC) in the freshly isolated j/aHDMEC, which were characterized by the different expression levels of podoplanin, namely LEC Podo^High^ (25.4 ± 7.2%) as well as LEC Podo^Low^ (10.5 ± 2.4%) (Figure 2a). Further analysis showed that LEC Podo^High^ are almost entirely positive for CD200 (98.8 ± 15.6%) (Figure 2b,e), whereas LEC Podo^Low^ displayed a significantly reduced expression of CD200 (36.9 ± 5.1%) (Figure 2c,e). 

Freshly isolated j/aHDMEC were further cultured in vitro and flow cytometric analyses regarding CD200 expression were repeated on HDMEC passage 1 (P1, cells after first passage) cells. Accordingly, we identified approximately 66.7 ± 16.2% of BEC and 33.3 ± 7.5% of LEC in cultured j/a P1 HDMEC. Further, we identified only a weak expression of CD200 on BEC (11.3 ± 2%), whereas the expression of CD200 on LEC was significantly enhanced and accounted for 48.7 ± 17.2% (*p* < 0.0001) (Figure 2i). The LEC Podo^Low^ population was lost in cultured LEC already at P1. 

Due to the small size of human fetal skin biopsies and, thus, low numbers of HDMEC in the total dermal cell mixture, the flow cytometry analysis of freshly isolated fetal endothelial cells was not possible. The analysis of cultured fetal P1 HDMEC revealed that fBEC (fetal BEC) constituted approximately 58.9 ± 12.5% of all endothelial cells and that only 9.06 ± 2.0% of fBEC showed the expression of CD200 (Figure 2j–m). By contrast, fLEC (fetal LEC) represented approximately 41.1 ± 9.3% of all fHDMEC and 51.9 ± 15.7% of those cells expressed CD200 marker (Figure 2j–m). Thus, the CD200 expression was significantly enhanced on fLEC as compared to fBEC (*p* < 0.0001) (Figure 2m).

The presence of the two subpopulations, namely LEC Podo^High^ and LEC Podo^Low^, were almost undetectable on cultured (in vitro expanded) j/a and fHDMEC at P1 in vitro, and, therefore, not further analyzed.

### 3.3. CD200^+^ LEC Demonstrate Higher Proliferation Rate Than CD200^−^ LEC and BEC In Vitro

**Figure 3:**
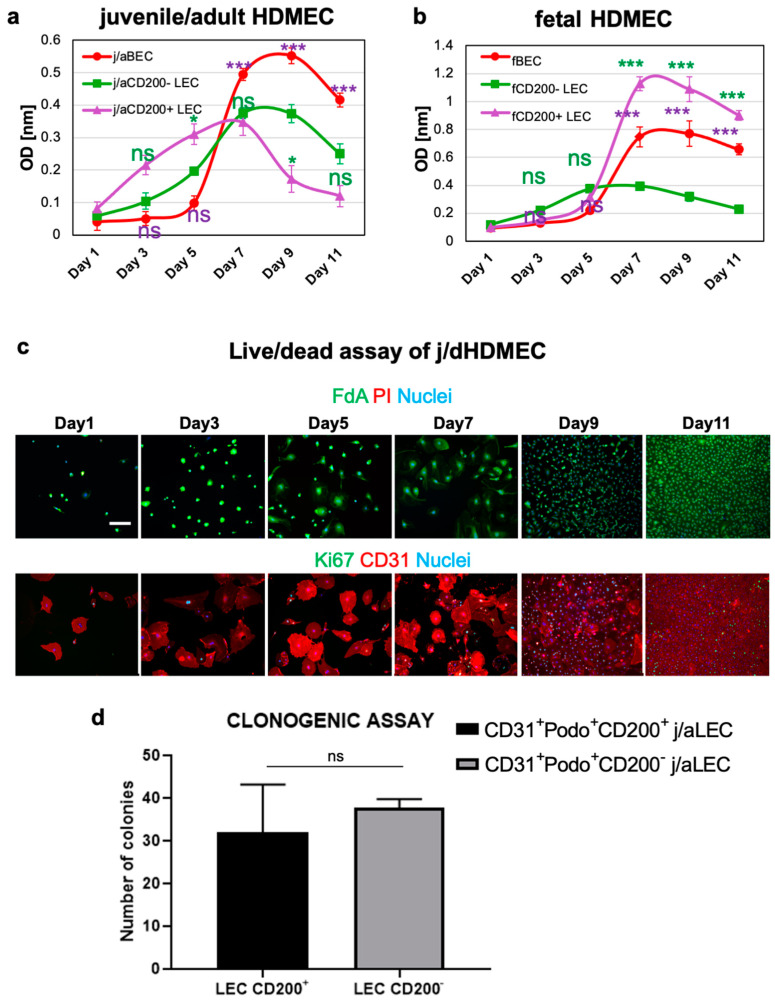
Proliferation rate of juvenille/adult (j/a) and fetal (f) CD200^+/−^ LEC and BEC in vitro. (**a**) Graphical representation of proliferation rates of CD200^+^ and CD200^−^ BEC and LEC isolated from j/a skin samples. Cells were plated at a concentration of 5 × 10^3^ cells per well (6-well plate), and their numbers were determined up to 11 days after plating by colorimetric cell counting assay. J/aCD200^+^ LEC (violet line) showed a higher proliferation rate in the first 5 days of the measurement as compared to j/aCD200^−^ LEC (green line) and j/aBEC (red line). In turn, from day 7–11 during the assay, j/aBEC showed the highest cycling rate. At the same time, j/aCD200^+^ LEC showed lower proliferation rate as j/aCD200^−^ LEC. Two-way ANOVA (analysis of variance) comparison of those two cell populations revealed following significance: d1/d5: *p* > 0.05 (ns); d3: *p* < 0.05; d7–d11: *p* < 0.001), whereas the comparison of j/aBEC vs. j/aCD200^+^ LEC demonstrated following values: d1/d3: 0.05 (ns); d5: *p* < 0.01; d7–d11: *p* < 0.001. (**b**) In days 1–5 of the assay, fCD200^+^ LEC (violet line) isolated from fetal skin revealed a slightly slower proliferation rate of as compared to fCD200^−^ LEC (green line; d1/d5: *p* > 0.05 (ns); d3: *p* < 0.05), however it was higher as compared to fBEC (d1–d3: *p* > 0.05 (ns); d5 *p* < 0.01). Further, from day 5–11 the fCD200^+^ LEC population demonstrated the highest cycling rate as compared to fCD200^−^ LEC (d5: *p* > 0.05 (ns); d7–d11: *p* < 0.001) and to fBEC (d5: *p* < 0.01; d7–d11: *p* < 0.001). Thus, fBEC showed a moderate proliferation rate with cycling values ranging between fCD200^+^ LEC and fCD200^−^ LEC within days 5–11 of the assay. A representative experiment out of *n* = 6 (j/a) and *n* = 3 (f) is shown (*n* = different biological samples performed in triplicates). Two-way ANOVA with Bonferroni multiple comparisons test with ns = not significant (*p* > 0.05); * for *p*-value 0.01 to 0.05 (significant); *** for *p* < 0.001 (extremely significant) was used for statistical analysis (green means CD200^+^ LEC/CD200^−^ LEC comparison; violet means CD200^+^ vs. BEC comparison). (**c**) Live-dead assay assessing viability at a 2-day interval in primary j/aHDMEC containing BEC/LEC. The data shows high cell viability even after confluence is reached in monolayer HDMEC culture. (**d**) Clonogenic assay of CD200^+^ and CD200^−^ lymphatic endothelial cells (LEC). Quantification of the number of colonies revealed that CD31^+^Podo^+^CD200^+^ formed 32 ± 11.0 and CD31^+^Podo^+^CD200^−^ 37.7 ± 2.08 (*p* = 0.4351, ns) lymphatic endothelial cells formed similar numbers of colonies 14 days after cell seeding. *n* = 3 independent skin donors each. Experiments were conducted in triplicates. Scale bar 100 μm.

CD200^+^ LECs and CD200^−^ LECs as well as donor-matched BEC derived and cultured from j/a and f skin were separated by fluorescence activated cell sorting (FACS) and used for colorimetric cell counting assay in vitro (Figure 3a). Proliferation assay using cells derived from j/a skin samples revealed the highest proliferation rate of j/aCD200^+^ LEC in the first 5 days of the analysis (Figure 3a, violet line) as compared to j/aCD200^−^ LEC (green line; d1/d5: *p* > 0.05 (ns); d3: *p* < 0.05) and BEC (red line; j/aBEC vs. j/aCD200^+^ LEC: d1/d3: 0.05 (ns); d5: *p* < 0.01). Interestingly, from day 7–11 during the proliferation assay, the population of j/aCD200^+^ LEC demonstrated a lower cycling rate as compared to j/aCD200^−^ LEC and j/aBEC. Two-way ANOVA comparison demonstrated the following significance: d7–d11: *p* < 0.001 (j/aCD200^+^ vs. j/aCD200^−^ LEC) and d7–d11: *p* < 0.001 (j/aBEC vs. j/aCD200^+^ LEC).

The proliferation assay of HDMEC isolated from fetal skin revealed a slightly slower proliferation rate of fCD200^+^ LEC (violet line) as compared to fCD200^−^ LEC (green line), whereas fCD200^+^ LEC proliferated faster than fBEC in days 1–5 of the assay (Figure 3b). In contrast, from day 5–11 the fCD200^+^ LEC population demonstrated the highest cycling rate as compared to fCD200^−^ LEC (d5: *p* > 0.05; d7–d11: *p* < 0.001) and to fBEC (d5: *p* < 0.001). Thus, fBEC showed a moderate proliferation rate with cycling values ranging between fCD200^+^ LEC and fCD200^−^ LEC within days 5–11 of the assay. 

Cell viability stainings performed on primary j/aHDMECs containing both BEC and LEC populations showed high cell viability throughout the 11-day culturing period (Figure 3c). In addition, Ki67 staining indicates that the fraction of Ki67+ cells was at highest at day 9, after which the number of proliferative cells decreased again (Figure 3c). Thus, metabolic activity is a leading indicator of proliferation, preceding cell division.

Further, Wakabayashi et al. identified CD200^+^ as one of the markers of mouse tissue-resident vascular endothelial stem cells with enhanced clonal expansion potential [40]. To investigate those characteristics in human j/a CD200^+^ and CD200^−^ LEC used in this study, we performed colony-forming assays (Figure 3d). The quantification revealed that j/aCD200^+^ LEC formed 32 ± 11.0 of colonies and j/aCD200^−^ LEC 37.7 ± 2.08 colonies on collagen I-coated dishes. Accordingly, both j/aCD200^+^/CD200^−^ LEC fractions demonstrated a similar colony-forming ability (*p* = 0.4351).

### 3.4. Specific Adhesion Molecules, Lymphatic Markers, and Chemokines Are Differently Expressed in Distinct HDMEC Populations In Vitro

**Figure 4:**
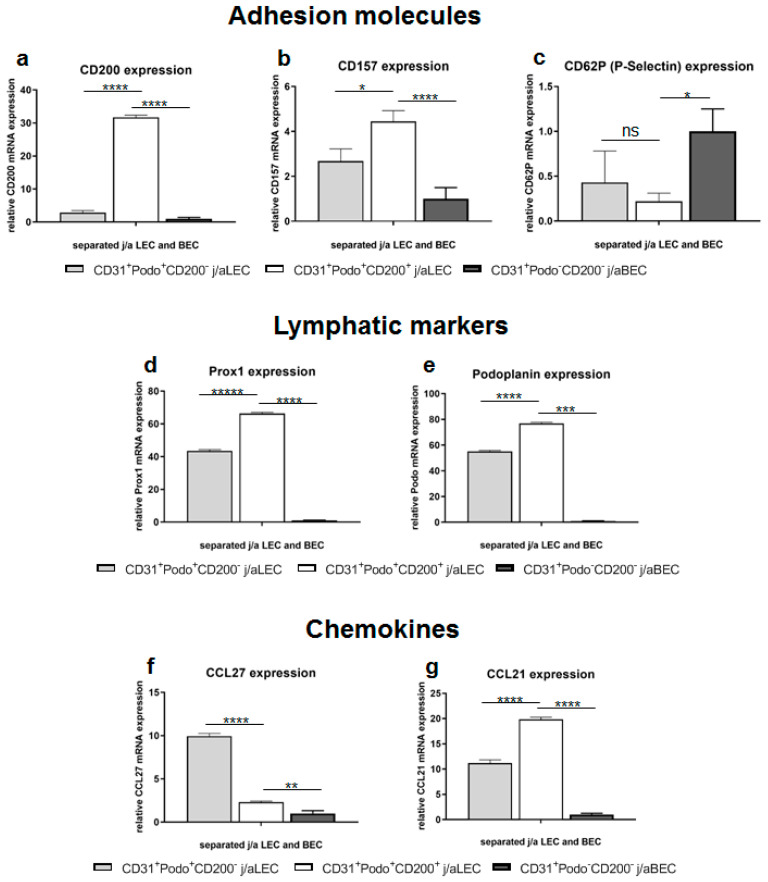
Gene expression analysis in distinct LEC and BEC fractions. HDMEC were harvested from j/a skin, cultured, separated at P0 into CD200^−^/CD200^+^ LEC and CD200^−^ BEC fractions and used directly for qRT-PCR. The relative gene expression levels were normalized to GAPDH housekeeping gene and displayed relative to CD200^−^ BEC samples. (**a**–**c**) Analysis of adhesion molecules confirmed 11-fold upregulation of CD200 mRNA in j/aCD200^+^ LEC (31.70 ± 0.67) as compared to j/aCD200^−^ LEC (2.88 ± 0.6; *p* < 0.0001) and j/aCD200^−^ BEC (1 ± 0.41; *p* < 0.0001) (**a**). Another adhesion molecule, CD157 was also enriched in j/aCD200^+^ LEC (4.44 ± 0.48) as compared to j/aCD200^−^ LEC (2.68 ± 0.54; *p* = 0.0107) and j/aCD200^−^ BEC (1 ± 0.5; *p* = 0.0003) (**b**). The expression of CD62P selectin mRNA was reduced in j/aCD200^+^ LEC (0.22 ± 0.09) in comparison to j/aCD200^−^ LEC (0.43 ± 0.35; *p* = 0.5773, ns) and j/aCD200^−^ BEC (1.0 ± 0.25; *p* = 0.0186) (**c**). (**d**,**e**) Assessment of mRNA expression of lymphatic markers. J/aCD200^+^ LEC showed upregulated expression of PROX1 (66.34 ± 0.74) and podoplanin (76.93 ± 0.77) as compared to j/aCD200^−^ LEC (PROX1: 43.55 ± 0.78, podoplanin: 55.13 ± 0.76; both *p* < 0.0001) and j/aCD200^−^ BEC (PROX1: 1 ± 0.41, podoplanin: 1 ± 0.09; both *p* < 0.0001). (**f**,**g**) Chemokines CCL27 and CCL21 showed various mRNA expressions, whereas CCL27 mRNA was downregulated in j/aCD200^+^ LEC (2.32 ± 0.11), CCL27 mRNA was upregulated in j/aCD200^−^ LEC (9.96 ± 0.30; *p* < 0.0001) as compared to j/aCD200^−^ BEC expression (1 ± 0.33; *p* = 0.0018). In contrast, CCL21 was significantly upregulated in j/aCD200^+^ LEC (19.86 ± 0.41), whereas CCL27 showed reduced mRNA levels in j/aCD200^−^ LEC (11.17 ± 0.66; *p* < 0.0001). Each bar represents mean ± SD of *n* = 3 biological samples. Experimental groups were compared using one-way ANOVA with Turkey’s multiple comparisons test. Asterisks denote significance as follows: * *p* < 0.05; ** *p* < 0.01; *** *p* < 0.001; and **** *p* < 0.0001.

To determine the gene expression in different j/aHDMEC populations, cells were harvested, cultured, separated at P0 into CD200^−^/CD200^+^ LEC and CD200^−^ BEC fractions, and used for qRT-PCR analysis. Gene expression levels were normalized to GAPDH and expression levels of each sample were calculated relative to the CD200-BEC population. We did not use fibroblasts at all for any kind of normalization or in general in this analysis (Figure 4). 

Notably, we confirmed the 11-fold enhanced mRNA expression of CD200 in j/aCD200^+^ LEC (31.70 ± 0.67) as compared to j/aCD200^−^ LEC (2.88 ± 0.6; *p* < 0.0001) and j/aCD200^−^ BEC (1 ± 0.41; *p* < 0.0001) (Figure 4a). Another adhesion molecule, CD157 was also enriched in j/aCD200^+^ LEC (4.44 ± 0.48) as compared to j/aCD200^−^ LEC (2.68 ± 0.54; *p* = 0.0107) and j/aCD200^−^ BEC (1 ± 0.5; *p* = 0.0003) (Figure 4b). However, the expression of CD62P selectin mRNA was reduced in j/aCD200^+^ LEC (0.22 ± 0.09), whereas it was enhanced in j/aCD200^−^ LEC (0.43 ± 0.35; *p* = 0.5773, ns) and normalized to j/aCD200^−^ BEC (1.0 ± 0.25; *p* = 0.0186) (Figure 4c). The expression of other adhesion molecules such as CD54, intercellular adhesion molecule 2 (ICAM2; CD102), vascular cell adhesion molecule 1 (VCAM-1; CD106), and junctional adhesion molecule A (CD321; F11R) did not show significant differences between different endothelial cell populations (data not shown).

Further, assessment of mRNA expression of PROX1 and podoplanin revealed upregulation of both markers in j/aCD200^+^ LEC (66.34 ± 0.74 and 76.93 ± 0.77, respectively) (Figure 4d,e). In contrast, PROX1 (43.55 ± 0.78; *p* < 0.0001) and podoplanin (55.13 ± 0.76; *p* < 0.0001) mRNA was significantly reduced in j/aCD200^−^ LEC, and the lowest expression was detected in j/aCD200^−^ BEC (PROX1: 1 ± 0.41; *p* < 0.0001, podoplanin: 1 ± 0.09; *p* < 0.0001). 

Moreover, we investigated the expression of chemokines like CCL21 and CCL27 on the mRNA level (Figure 4f,g). Whereas CCL21 was significantly upregulated in j/aCD200^+^ LEC (19.86 ± 0.41), reduced mRNA levels were observed in j/aCD200^−^ LEC (11.17 ± 0.66; *p* < 0.0001) and j/aCD200^−^ BEC (1 ± 0.25; *p* < 0.0001) (Figure 4f). In contrast, CCL27 mRNA expression was downregulated in j/aCD200^+^ LEC (2.32 ± 0.11), whereas CCL27 gene expression was upregulated in j/aCD200^−^ LEC (9.96 ± 0.30; *p* < 0.0001) relative to j/aCD200^−^BEC 1 ± 0.33 (*p* = 0.0018) (Figure 4g). 

### 3.5. CD200R Is Expressed on Distinct Subsets of Human and Rat Immune Cells 

As CD200R is involved in immunosuppressive activity during skin inflammation and was shown to improve graft take and survival [41], we sought to determine the expression of CD200R in distinct subsets of human and rat immune cells.

CD200R expression on immune cells isolated from human peripheral blood (PBMC) was first verified by FACS, and then respective cells were sorted (Supplementary Figure S4). Accordingly, granulocytes were gated by side scatter first, and then assessed for CD11b (integrin alpha M), CD15, and CD200R marker expression to separate a population of CD11b^+^CD15^+^CD200^+^ granulocytes [42]. This granulocyte fraction was also positive for pan-granulocyte marker HIS48 (data not shown). The analysis revealed that 70% ± 15.7 of human blood granulocytes demonstrated CD200R^+^ expression (*n* = 3 blood donors) (Supplementary Figure S4a,a’’).

Further, using low forward/side scatter and CD3 marker present at all stages of T-cell development, we identified a population of CD3^+^ T-cells in human blood PBMC [43] (Supplementary Figure S4b,b’’). The T cells demonstrated a high expression of CD200R, which accounted for 95% ± 22.5 of this subset. This subset also contained human natural killer T-cells (NKT) characterized by the co-expression of CD3^+^CD56^+^.

In addition, a human monocyte subset gated also on low forward/side scatter and on CD14 marker was identified in human blood PBMC (Supplementary Figure S4c,c’). In detail, we detected two populations of CD14^+^ blood cells: classical CD14^+^CD16^−^ and non-classical CD14^+^CD16^+^ monocytes that expressed CD200R. The mean expression of CD200R was similar on both classical CD14^+^CD16^−^ monocytes (75% ± 19.2) (Supplementary Figure S4c’’) and on non-classical CD14^+^CD16^+^ monocytes (70% ± 15.5).

Further, the distribution of CD200R was assessed on immune cells in rat lymph nodes. We detected the CD200R expression on rat myeloid cells using rat-specific pan-myeloid antibody (Supplementary Figure S9a–c). Moreover, HIS48-positive rat granulocytes and CD68-expressing monocytes/macrophages were also positive for CD200R in native rat lymph nodes (Supplementary Figure S9d–i, respectively).

### 3.6. Role of CD200 and CD200R in Endothelial-Immune Cell–Cell Interactions In Vitro

**Figure 5:**
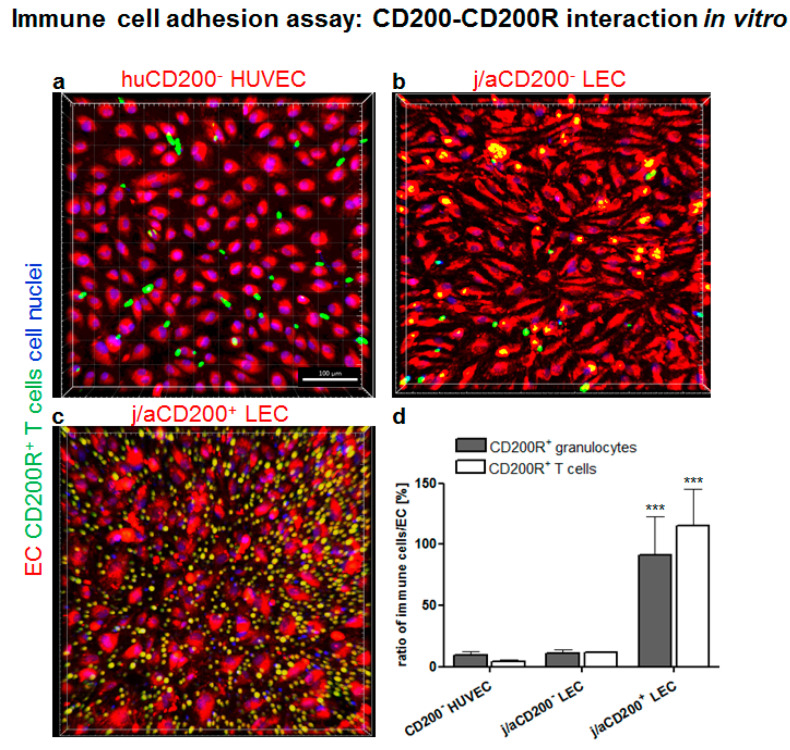
Cell adhesion assay of human immune cells to distinct endothelial cell (EC) populations. (**a**–**c**) HUVEC (CD200^−^) (**a**), j/aCD200^−^ (**b**), and j/aCD200^+^ LEC (**c**) (all pre-labeled red) were co-cultured with separated human blood-derived granulocytes and T-cells (all pre-labeled green). (**d**) Cell adhesion assay shows a decreased population of pre-labeled human granulocytes and T-cells cells adhering to both CD200^−^ cells: HUVEC (**a**) and j/aLEC (**b**) as compared to the high binding of both immune cell types to j/aCD200+ LEC (**c**) in vitro. (**d**) The quantification of the ratio of immune cells to EC confirmed that human blood-derived granulocytes and T-cells demonstrated specific adherence to j/aCD200^+^ LEC, whereas those immune cells did not show adherence to CD200^−^ EC. Accordingly, the assessed adhesion ratio of CD200R^+^ granulocytes (9.77 ± 2.58) and T-cells (4.27 ± 1.59; *p* = 0.99 (ns)) to HUVEC was low and similar to the binding ratio to j/aCD200^−^ LEC (11.11 ± 3.19 and 11.84 ± 0.93; *p* = 0.99 and *p* = 0.99 (ns), respectively). Further, the data showed that the adhesion rate of both immune cell types was significantly enhanced for j/aCD200^+^ LEC (91.27 ± 31.71 and 115.22 ± 29.69; *p* = 0.0005 and *p* < 0.0001, respectively). The results are expressed as mean ± SD from *n* = 6 biological samples of EC populations and *n* = 3 PBMC donors of granulocytes and T-cells. Asterisks denote significance as follows (unpaired *t*-test): *** *p* < 0.001. An unpaired *t*-test was used. Cell nuclei are stained with Hoechst (blue). Scale bars: 100 μm.

The interaction of the CD200 ligand with CD200R expressing immune cells was further investigated using a cell adhesion assay (Figure 5). Distinct EC populations like j/aCD200^+^ and CD200^−^ LECs and HUVEC were co-cultured with human blood-derived granulocytes and/or T cells and visualized using confocal microscopy. We confirmed that HUVEC were CD31^+^ but completely lacked the expression of podoplanin (Supplementary Figure S6a,a’), resembling the expression pattern of dermal BEC fraction. In addition, HUVEC lacked the expression of CD200 and, therefore, were used as a negative control for the cell adhesion assay (Supplementary Figure S5b,b’).

Initially, different endothelial cell fractions: j/aCD200+ and CD200- LEC and HUVEC, were labeled using red cell tracker (red) and co-cultured with human blood-derived granulocytes or T-cells labeled with a green cell tracker and co-cultured (Figure 5a–c). The quantification of the ratio of immune cells to endothelial cells demonstrated that only a few CD200R+ granulocytes (9.77 ± 2.5) and T-cells (4.27 ± 1.59; *p* = 0.99 (ns) adhered to HUVEC in the co-culture assay (Figure 5a). Similarly, only a few granulocytes (11.11 ± 3.19; *p* = 0.99) and T-cells (11.84 ± 0.93; *p* = 0.99) adhered to the j/aCD200^−^ LEC (Figure 5b) as compared to HUVEC. In contrast, a high number of CD200R-expressing granulocytes (91.27 ± 31.71; *p* = 0.0005) and T-cells (115.22 ± 29.69; *p* < 0.0001) adhered specifically to j/aCD200^+^ LEC (Figure 5c) as compared to j/aCD200^−^ LEC. The results show evidence that CD200R-expressing immune cells demonstrated specific adhesion only to monolayers of endothelial cells expressing CD200 ligand, whereas almost no binding was detected using CD200^−^ endothelial cells. Furthermore, there was no significant difference between the adhesion of granulocytes and T-cells to different EC fractions. 

Involvement of endothelial CD200 in the cell–cell interactions between immune cells (blood-derived T-cells) and the endothelium was further investigated by pre-incubation of primary HDMEC with anti-CD200 antibody to block the CD200 epitopes (Supplementary Figure S6). IgG1 treated and untreated HDMEC were used as a negative control. CD200 antibody treatment revealed that the adhesion of blood T-cells on the HDMEC monolayer was almost totally blocked by the antibody pre-incubation with the endothelial cells (9.4% ± 3.4), whereas IgG treatment (110.8% ± 27.6) and no treatment (119.7% ± 14.2) demonstrated significantly higher immune-cell binding. The blocking efficiency of CD200 was verified using immunofluorescence staining of adherent HDMEC previously blocked with unconjugated CD200 and subsequently stained with conjugated CD200 of the same clone (Supplementary Figure S7).

### 3.7. CD200 Is Expressed on Cultured Capillaries in 3D Hydrogels In Vitro

**Figure 6:**
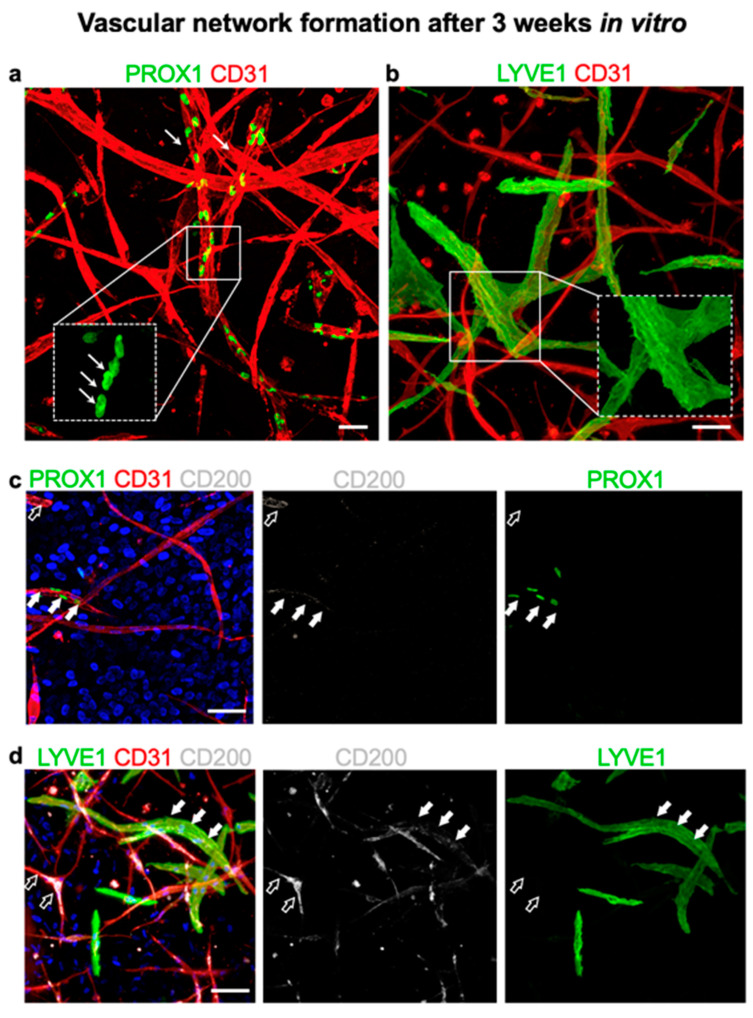
Expression of CD200 on in vitro cultured blood and lymphatic capillaries in 3D collagen. (**a**) Collagen type I-based hydrogel, and stained for endothelial-specific marker CD31 (red) and lymphatic lineage marker PROX1 (green) (*n* = 5). Whereas double-positive CD31^+^PROX1^+^ capillaries represent lymphatics, single positive CD31^+^PROX1^−^ demonstrate blood capillaries. The lower inset shows a higher magnification of the single nuclear PROX1 staining in lymphatic capillaries (arrows). (**b**) The merged confocal immunofluorescence showing CD31^+^ blood capillaries (red) and lymphatic capillaries positive for CD31^+^ (red) and LYVE1 (green), which is a lymphatic-specific marker. Insets show a magnification of single-stained LYVE1 lymphatic capillaries. (**c**) Triple stained image of human capillary network co-stained for PROX1^+^ (green) CD200^+^ (white) CD31^+^ (red). Accordingly, PROX1^+^CD200^+^CD31^+^.triple positive capillaries represent lymphatics expressing CD200 marker (filled arrows), whereas double-positive PROX1^−^CD200^+^CD31^+^ staining demonstrates blood capillaries positive for CD200 (empty arrows). (**d**) Triple co-staining for LYVE1^+^ (green) CD200^+^ (white) CD31^+^ (red). Whereas the majority of lymphatic capillaries (CD31^+^LYVE1^+^) were positive for CD200 (filled arrow), only a few blood capillaries (CD31^+^LYVE1^−^) expressed CD200 (empty arrows). The results are representative from *n* = 3 biological samples of EC populations. Cell nuclei are stained with Hoechst (blue). Scale bars: (**a**–**d**): 50 μm.

To analyze the expression of CD200 on capillaries cultured in vitro, we used a 3D collagen I hydrogel-based system (Figure 6a–d). J/a human dermal microvascular endothelial cells (HDMEC) co-culture with dermal fibroblasts developed spontaneously into 3D vascular networks in vitro. Notably, we were able to reproduce the physiological ratio of approximately 70:30% of lymphatic to blood capillaries in vitro as demonstrated by CD31 and Prox1 or LYVE1 markers using immunofluorescence whole-mount stainings of 3D hydrogels (Figure 6a,b). Furthermore, we detected CD200 expression on both blood (CD31^+^PROX1^−^) and lymphatic (CD31^+^PROX1^+^) capillaries (Figure 6c, respectively). However, similar to the j/a human skin, the expression of CD200 was enhanced on lymphatic as compared to blood capillaries.

These findings were conformed using another lymphatic marker, namely LYVE1 (Figure 6b,d). A triple co-staining for LYVE1, CD200, and CD31 demonstrated that the majority of lymphatic capillaries (CD31^+^LYVE1^+^) were positive for CD200, whereas only a few blood capillaries (CD31^+^LYVE1^−^) expressed CD200.

### 3.8. CD200 Is Expressed on Capillaries of Human Prevascularized DESS In Vivo 

**Figure 7:**
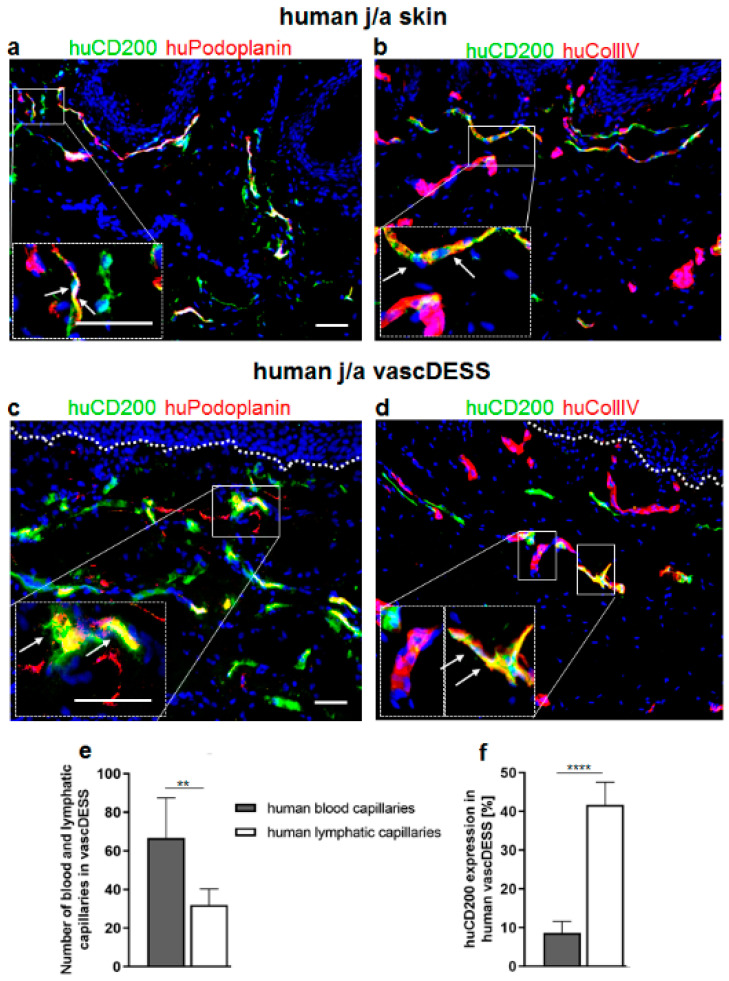
Quantification of CD200 expression in transplanted vascDESS. (**a**,**b**) Immunofluorescence co-staining of j/a human skin against huCD200 (green) and huPodoplanin (**a**) or collagen IV (**b**). Podoplanin depicts human lymphatic endothelium, while collagen IV is expressed exclusively around human blood capillaries. White arrows in magnification insets indicate double-positive microvascular structures. (**c**,**d**) Immunoflorescence co-staining of vascDESS against huCD200 (green) as well as huPodoplanin (red), visualizing the human lymphatic capillaries (**c**) and huCollagen IV (red) depicting the human blood endothelium (**d**). (**e**) Quantification revealed that vascDESS contains a significantly higher number of blood (66.59 ± 20.95) than lymphatic capillaries (32 ± 8.34; ** *p* = 0.009). Presented values are the mean (±SD) of total numbers of human CD31^+^ or podoplanin^+^ vessels counted per 10× high-power field, *n* = 6 biological samples in each group. White arrows in magnification insets indicate double-positive microvascular structures. (**f**) Quantification of CD200 expression on blood and lymphatic vessels in vascDESS. Please note that CD200 is expressed mainly on the lymphatic vessels (41.67 ± 5.89), while blood vessels exhibit only minor expression of that marker (8.63 ± 2.95; **** *p* < 0.0001). The results are expressed as mean ± SD from *n* = 3 biological donors from 3 independent animal transplantation (6 animals in total). Cell nuclei are stained with Hoechst (blue). Scale bars 50 μm.

As demonstrated on microvasculature in human skin, CD200 is specifically expressed on lymphatic rather than on blood capillaries (Figure 1 and Supplementary Figure S8). Since podoplanin (Podo) expression was restricted to lymphatics (Figure 1), we used podoplanin to delineate specifically lymphatic capillaries (Figure 7a and Supplementary Figure S8b) and IV (Coll IV) to detect only blood capillaries of human j/a skin and DESS in vivo (Figure 7b and Supplementary Figure S7c). In contrast to lymphatic endothelia, blood capillaries demonstrate a continuous basement membrane expressing Coll IV (Figure 7b and Supplementary Figure S7c). 

Further, using those markers, we assessed the expression of CD200 on human lymphatic and blood capillaries of vascDESS in vivo (Figure 7c–d,f). Human vascDESS demonstrated a heterogeneous distribution pattern of human CD200, which was restricted to capillaries in the dermis, similar to the pattern observed in normal human skin (Figure 7a–d). The quantification performed three weeks after transplantation revealed that CD200 was highly expressed on human podo-positive lymphatic capillaries (41.67 ± 5.89), whereas it was only scarcely detected on human Coll IV-positive blood capillaries (8.63 ± 2.95; *p* < 0.0001) (Figure 7c–d,f). Thus, like in human skin, CD200 showed a 4-fold higher expression on lymphatic as compared to blood capillaries in vascDESS. 

### 3.9. CD200 Binds to Its Cognitive Receptor, CD200R in Human Skin and Skin Substitutes In Vivo 

Involvement of human endothelial CD200 in the cell–cell interactions between the endothelium and CD200R^+^ immune cells has not been investigated so far in human skin. 

**Figure 8:**
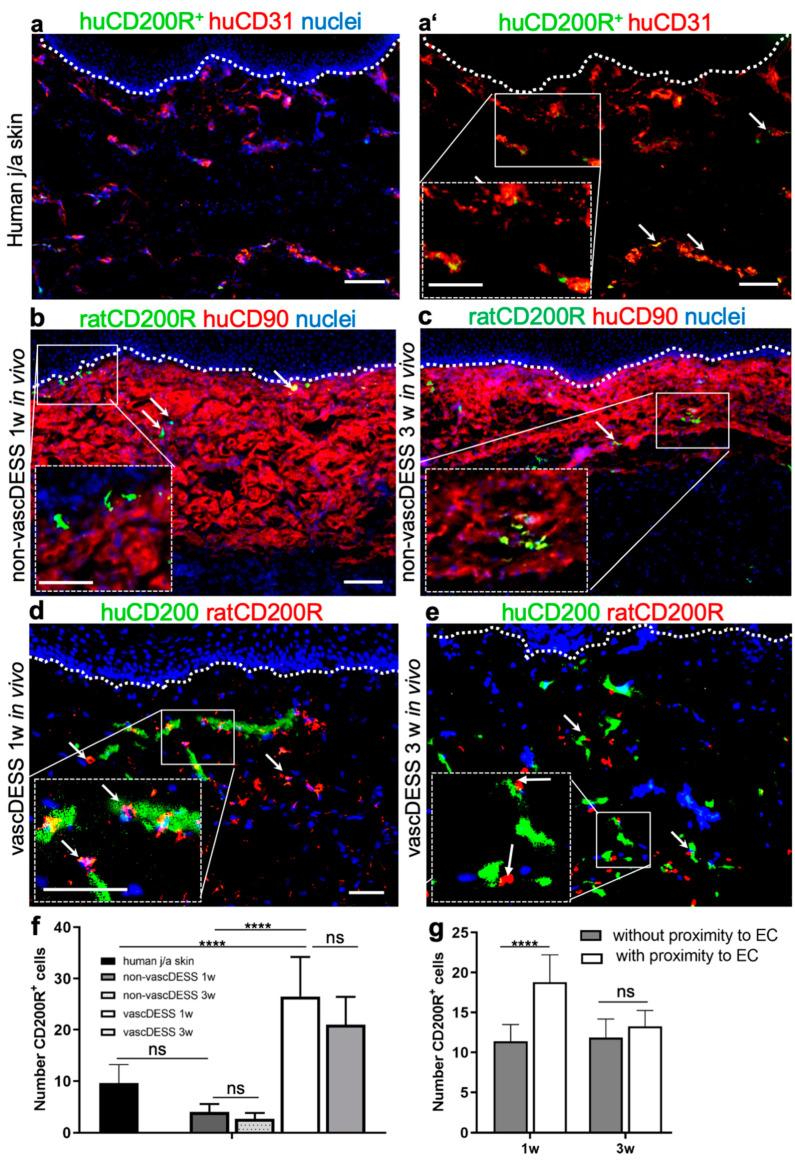
Expression of CD200R in human j/a skin as well as in non-vascDESS and vascDESS. (**a**,**a’**) Immunofluorescence pictures of j/a skin stained against CD31 (red) and CD200R (green). Note that CD200R^+^ immune cells (9.65 ± 3.56) are closely associated with CD31^+^ capillaries. (**b**,**c**) The expression of CD200R in non-vascDESS at one week (4 ± 1.55, *p* = 0.1551 vs. normal skin) (**b**) and 3 weeks (2.67 ± 1.55, *p* = 0.0565 vs. normal skin) (**c**) after transplantation. Once again, CD200R-expressing cells were located in close proximity to the CD31^+^ capillaries. (**d**,**e**) The expression of CD200R in vascDESS one week (26.5 ± 7.71, *p* < 0.0001 vs. non-vascDESS 1w, **** *p* < 0.0001 vs. human skin) (**d**) and three weeks (21.31 ± 5.4, *p* < 0.0001 vs. non-vascDESS 1w, *p* < 0.0001 vs. human skin) (**e**) post-transplantation. Numerous CD200R^+^ cells were visible throughout the entire dermal layer and adhered mainly to the CD200^+^ endothelium. (**f**) Quantification of CD200R-expressing cells in the dermal compartments of j/a human skin as well as in non-vasc and vascDESS after one and three weeks in vivo. Please note the significantly higher number of CD200R^+^ cells in the dermis of vascDESS, as compared to the native skin or non-vascDESS. In contrast, human vascDESS containing human blood and lymphatic capillaries demonstrated enhanced density of rat CD200R-positive cells at one (26.5 ± 7.71, *p* < 0.0001 vs. non-vascDESS one week, **** *p* < 0.0001 vs. human skin) and three (21.31 ± 5.4, **** *p* < 0.0001 vs. non-vascDESS 1w, *p* < 0.0001 vs. human skin) weeks in vivo (**d**,**e**). White arrows in magnification insets indicate CD200–CD200R interactions. (**g**) In vivo, significantly more CD200^+^ endothelial cells were associated with rat CD200R^+^ immune cells 1 w post-transplantation compared to CD200R^+^ cells that were non-associated with CD200^+^ endothelial cells (**** *p* < 0.0001). This difference disappears after 3 w in vivo (ns, *p* > 0.05). The results are expressed as mean ± SD from *n* = 3 biological donors from 3 independent animal transplantation (6 animals in total). Cell nuclei are stained with Hoechst (blue). Scale bars 100 μm and 50 μm (magnification insets).

Therefore, we sought to determine the spatiotemporal interaction between CD200R-expressing cells with CD200 in normal human skin, non-vascularized DESS (control), and vascularized DESS (vascDESS) at one and three weeks after transplantation (Figure 8). Further, we have confirmed that rat CD200R^+^ cells were of myeloid origin, thus co-expressing myeloid, granulocytes, and monocyte/macrophage-specific markers in rat tissue (Supplementary Figure S4).

Whereas only a few CD200R-positive cells were detected in normal human j/a skin 9.65 ± 3.56 (Figure 8a,a’,f), most CD200R-expressing cells were located in close proximity to capillaries as demonstrated by a co-staining for human CD31 (Figure 8a,a’, arrows). 

Similar, non-prevascularized (non-vasc) DESS also showed only a scarce number of CD200R^+^ cells within the human dermal compartment at one week (4 ± 1.55, *p* = 0.1551 vs. normal skin) and at three weeks (2.67 ± 1.55, *p* = 0.0565 vs. normal skin) after transplantation (Figure 8b–d). 

In contrast, human vascDESS containing human blood and lymphatic capillaries demonstrated enhanced density of rat CD200R-positive cells at 1 week (26.5 ± 7.71, *p* < 0.0001 vs. non-vascDESS 1w, *p* < 0.0001 vs. human skin) and three weeks (21.31 ± 5.4, *p* < 0.0001 vs. non-vascDESS 1w, *p* < 0.0001 vs. human skin) in vivo (Figure 8d,e). Although dermal cells positive for CD200R were distributed throughout the entire dermal layer (Figure 8d), the major subset of those cells adhered to CD200-positive endothelia (Figure 8d,e; arrows), demonstrating an active CD200–CD200R axis in vascDESS at 1 and 3 weeks post-transplantation. In particular, after 1w of in vivo culturing time, significantly more CD200R^+^ cells were associated with CD200^+^ endothelial cells compared to cells that were not in close proximity (18.8 ± 3.4 vs. 11.4 ± 2.1, *p* < 0.0001). This difference disappeared and there was a similar number of CD200R^+^ T cells was found associated (13.3 ± 2.0) and non-associated with CD200-positive endothelial cells (11.9 ± 2.3, *p* = 0.2064).

## 4. Discussion

Until today, the distribution of endothelial CD200 and its interaction with CD200R has not been reported in human skin and tissue-engineered dermo-epidermal skin substitutes. In particular, we have shown that: (i) CD200 was differently expressed in blood and lymphatic capillaries, (ii) CD200R was primarily expressed by myeloid and T-cells, (iii) CD200R on myeloid cells interacts with its cognate CD200 ligand expressed on endothelial in vitro and in vivo. Overall, our study demonstrated that the active CD200–CD200R axis plays a crucial role during wound healing of skin substitutes in vivo. Certain aspects of the study deserve more careful consideration.

We found that CD200 was abundantly expressed on small capillaries in normal human skin and bioengineered human vascDESS in vivo, in particular on lymphatic rather than blood capillaries of human skin and adipose tissue. This is in contrast to the study of Ko et al. that examined the distribution of endothelial CD200 in different types of vessels, including small capillaries and large-diameter vessels in different rat organs except for skin [19]. Whereas the authors described a pronounced CD200 immunoreactivity in small blood capillaries and almost no signal in arterioles and arteries, double labeling for CD200 and podoplanin showed rather weak or undetectable expression on lymphatic endothelia [19]. Interestingly, in our study, we detected an increased expression of CD200 on lymphatic endothelium in human skin and vascDESS as compared to blood capillaries. Thus, our findings might indicate that CD200 expression has some specific functions within human lymphatics. Whereas the relative expression of CD200 on lymphatic capillaries in fetal skin was found to be comparable to the levels found in j/a skin, fetal blood capillaries showed a markedly higher CD200 expression than their j/a counterpart. Using FACS on freshly isolated j/aHDMEC, we were able to confirm the relative CD200 expression levels in j/aBEC and j/aLEC also on a single-cell level. Re-analysis of previously unsorted HDMEC after a short culturing time (1 passage) revealed a slight decrease in relative CD200 expression on j/aLEC and fLEC. In contrast, only minor changes were observable in j/a BEC.

Moreover, Ko et al. [19] reported the distribution of CD200 protein in the rat endothelia of veins and venules in vivo in the bone marrow and the spleen, whereas, in our study, we did not observe CD200 on macrovascular EC derived from veins such as HUVEC on the protein and mRNA level. This discrepancy may be due to obvious differences between rat and human skin and, therefore, it is not possible to directly compare the results of those two studies. However, to the best of our knowledge, there is no report regarding CD200 distribution in human skin.

Another investigation performed by Wakabayashi et al. in mice detected the CD200 expression in ECs of most organs, except in the liver [40]. Moreover, the authors determined that CD200 was also co-expressed on all CD157^+^ ECs. Interestingly, CD157 was characterized in their study as a marker of tissue-resident vascular endothelial stem cells (VESC) with higher proliferative potential and clonal expansion than CD157^−^CD200^−^ ECs [40]. The mouse VESC described by Wakabayashi et al. were characterized by their increased proliferation rates as well as an enhanced colony-forming potential and their ability to differentiate into mature EC during wound healing [40]. 

Although the authors did not investigate CD200 expression on human ECs, nor did they distinguish between mouse blood and lymphatic endothelial cells [40], their study implies that CD200 might serve as a putative vascular stem cell marker also in human ECs. Thus, we performed clonogenic and proliferative assays in our study to assess a possible stem cell potential of CD200^+^ EC. However, we could not observe enhanced homeostatic and regenerative stem cell properties using CD200^+^ as compared to CD200- EC suggesting another possible function of CD200^+^ cells in human tissues. Therefore, further investigations are needed to validate the potential role of CD200^+^ human microvascular ECs as VESC in human skin.

It has been demonstrated that the specific expression of surface molecules is responsible for the trafficking of T-cells, monocytes/macrophages, and other leukocytes on the endothelium during tissue inflammation [44,45]. Further, pronounced CD200 immunoreactivity has also been previously reported on the specialized endothelium of the high endothelial venule (HEV) in the rat lymph nodes [19]. HEV forms branching networks of highly spatially organized post-capillary venules recruiting and controlling the entrance of lymphocytes from the bloodstream into lymph nodes. Following stimulation by antigen, activated lymphocytes exit the lymph node via lymphatics and re-enter the bloodstream [46]. Thus, the pronounced endothelial CD200 expression detected in HEV indicates that CD200–CD200R interaction may be involved in the regulation of immune cell trafficking in vivo.

Additionally, we demonstrated the highest expression of CD200 on Podo^High^ lymphatic endothelial cells (LEC), whereas Podo^Low^ LEC barely expressed this marker. Interestingly, Podo^High^ LEC are restricted to lymphatic precollector vessels, whereas Podo^Low^ LEC are exclusively lining lymphatic capillaries. The heterogeneous distribution of endothelial CD200 among different sites of the lymphatic vascular bed indicates a possible function of CD200^+^ Podo^High^ lymphatic capillaries in the formation and maintaining of HEV. Indeed, previous studies demonstrated the crucial role of podoplanin expression in HEV integrity in an immune cell trafficking into the lymph nodes [47,48].

Thus, the data obtained in this study indicate that CD200–CD200R interaction is critical for the proper function of the innate immune system by providing communication between CD200^+^ ECs and CD200R-expressing immune cells in HEV. 

Further, ECs lining human skin microvasculature are the first contact with most blood-borne cells, such as immune cells within the skin. In the present study, we examined the expression and distribution of the CD200R, which is a cognate receptor of CD200. We found CD200R on monocytes/macrophages, granulocytes, and T-cells in both rat and human skin as well as in tissue-engineered DESS. As previously reported CD200 binding to CD200R expressed mainly on CD11b^+^ myeloid cells, monocytes/macrophages, and dendritic cells repress their pro-inflammatory activation and interleukin production in vivo [14,15,49,50,51]. This specific interaction inhibits MAP kinases p38, ERK, and JNK, the common signaling pathways involved in the classic activation of macrophages, theoretically maintaining anti-inflammatory M2 cells in their polarized state [52]. Further, previous studies in mice reported that the application of a specific anti CD200-immunoglobulin also induced immune tolerance in dendritic cells [53]. 

Those results suggest that CD200–CD200R physical binding may have an important role and interactions in cell adhesion between immune cells and endothelium [19]. We confirmed here that CD200 expression in the skin microvasculature is indeed required for the specific adhesion of blood-derived monocytes/macrophages and T-cells in vitro. These data are in line with previous reports showing that anti-human CD200 antibodies completely blocked the interaction between human T cells (Jurkat T cells) and ECs in vitro [19]. Thus, together with previous reports, the data obtained in this study suggest a specific role of CD200–CD200R in the downregulation of the immune system by blocking activated monocytes/macrophages and T-cells.

Further, we detected a specific CD200–CD200R interaction in vascDESS in vivo. In particular, mainly lymphatic endothelial CD200^+^ interacted with CD200R. These data are in line with the observations of Ko et al. that showed that CD200–CD200R signaling mediates cell to cell interactions of immune cells with ECs in mouse tissues in vivo [19]. The same group also reported that CD200–CD200R signaling prevents further diapedesis in mouse in vivo, and thus, it reduces tissue inflammation following injuries [19]. Moreover, as described by Ngwa et al., the CD200–CD200R signaling leads to potent immunosuppression upon inflammatory stimuli [54]. Further, CD200 overexpression was shown to exert a protective function in autoimmune inflammation in models of autoimmune encephalomyelitis (EAE) [15], autoimmune uveoretinitis (EAU) [22], collagen-induced arthritis [55], and inflammation-mediated neurodegeneration [56]. 

Importantly, the interaction of CD200 with CD200R has been associated with a reduced risk of graft rejection as well as prolonged transplantation survival in mice [41]. CD200 was shown to be upregulated in mice transplantation models where successful inhibition of rejection is accomplished and is believed to signal immunosuppression following engagement of a receptor, CD200R, on myeloid cells [57]. Upon binding, CD200:CD200R mediates an alteration in cytokine production, with increased expression of anti-inflammatory cytokines: interleukin-4 (IL-4), IL-10 and transforming growth factor-β (TGF-β), and decreased IL-2, interferon-γ (IFN-γ) and tumor necrosis factor-α (TNF-α) [58]. Moreover, in vitro incubation of allostimulated cells in the presence of CD200 leads to inhibition of cytotoxic T-lymphocytes (CTL) reaction; an effect was also seen following in vivo engraftment [49].

Therefore, the interaction of CD200 with its CD200R is of pivotal importance for skin tissue engineering. As such, the incorporation of CD200^+^ EC into prevascularized skin substitutes could prevent excessive inflammation by its immune-suppressive function, thus reducing the risk of graft rejection [19]. Accordingly, we reported previously improved wound healing and graft take rate of vascDESS as compared to non-vascDESS [6,7,8,59,60]. In these studies, we previously demonstrated enhanced graft rejection, delayed graft take and prolonged pro-inflammatory M1 macrophage response in non-vascDESS compared to vascDESS, suggesting that the CD200–CD200R axis on EC/myeloid cells plays an important role in these outcomes [6,7,8,59,60] Our data are consistent with previous evidence from Hoek et al. that CD200 exerts an inhibitory function on myeloid cells [15], and that overexpression of CD200 signaling contributes to the polarization of cytokine production towards an anti-inflammatory, pro-regenerative cytokine profile [57]. 

## 5. Conclusions

In summary and conclusion, this appears to be the first report demonstrating the physical interaction of CD200–CD200R in human skin and transplanted skin grafts. The CD200 was present mainly on Podo^High^ lymphatic pre-collectors, suggesting its specific role in the regulation of immune cells trafficking. Importantly, CD200–CD200R is also highly active during wound healing of transplanted skin grafts. It appears likely that a similar interaction between CD200 and CD200R will take place when autologous tissue-engineered skin analogs are transplanted onto human patients. Further, these results implicate that the prevascularisation approach of skin substitutes allows proper cell–cell interactions in the wound bed leading to improved graft take and skin regeneration. Further investigation should explore the function of CD200R^+^ myeloid cells and their interaction with CD200 in human skin transplants in more detail.

## Figures and Tables

**Table 1:** Antibody conjugates used in flow cytometry.

Antibody Conjugate	Clone	Product Number	Manufacturer	Dilution
CD15-PE (SSEA-1)	W6D3	323006	BioLegend, Switzerland	1:20
PE Mouse IgG1, κ Isotype Ctrl	MOPC-21	400114	BioLegend, Switzerland	1:20
CD56-APC	MEM-188	304610	BioLegend, Switzerland	1:20
APC Mouse IgG2a, κ Isotype Ctrl	MOPC-173	400220	BioLegend, Switzerland	1:20
CD3-FITC	UCHT1	300452	BioLegend, Switzerland	1:50
FITC Mouse IgG1, κ Isotype Ctrl	MOPC-21	400108	BioLegend, Switzerland	1:50
CD14-FITC	M5E2	555397	BD Biosciences, Switzerland	1:50
CD200-AF647	OX-104	329214	BioLegend, Switzerland	1:50
Alexa Fluor 647 Mouse IgG1, κ Isotype Ctrl	MOPC-21	400130	BioLegend, Switzerland	1:50
CD200R-APC	OX-108	329308	BioLegend, Switzerland	1:20
APC Mouse IgG1, κ Isotype Ctrl	MOPC-21	400122	BioLegend, Switzerland	1:20
CD31-PE	WM59	555446	BD Biosciences, Switzerland	1:20
PE Mouse IgG1, κ Isotype Control	MOPC-21	555749	BD Biosciences, Switzerland	1:50
Podoplanin-AF488	NC-08	337006	BioLegend, Switzerland	1:50
Alexa Fluor^®^ 488 Rat IgG2a, κ Isotype Ctrl	RTK2758	400525	BioLegend, Switzerland	1:50
ZombieAqua Fixable Viability Kit	-	423102	BioLegend, Switzerland	1:500

**Table 2:** Antibodies used in immunohistochemical stainings.

Antibody	Clone	Product Number	Manufacturer	Dilution
**Anti-human**				
CD31	JC70A	M082301-2	Dako, USA	1:50
CD31-PE	WM59	555446	BD Biosciences, Switzerland	1:50
CD90	AS02	CP28-200UG	Merck Millipore, Germany	1:100
CD200	OX-104	329202	BioLegend, Switzerland	1:50
CD200-AF647	OX-104	329214	BioLegend, Switzerland	1:50
CD200R	OX-108	329302	BioLegend, Switzerland	1:50
CD200R-APC	OX-108	329308	BioLegend, Switzerland	1:20
Collagen IV	COL-94	ab6311	Abcam, UK	1:200
Ki67	B56	550609	BD Biosciences, Switzerland	1:100
LYVE1	polyclonal	Ab10278	Abcam, UK	1:100
Podoplanin	18H5	sc-59347	Santa Cruz, USA	1:100
Podoplanin-AF488	NC-08	337006	BioLegend, Switzerland	1:50
Podoplanin-AF647	NC-08	337008	BioLegend, Switzerland	1:20
PROX1	polyclonal	102-PA32S	ReliaTech, Germany	1:100
**Anti-rat**				
Anti-rat Granulocytes	HIS48	sc-19613	Santa Cruz, USA	1:100
Anti-rat Myeloid Lineage Antibody-FITC	OX-82	205103	BioLegend, Switzerland	1:50
CD68	PG-M1	Ab783	Abcam, UK	1:100
CD200R-FITC	OX-102	204905	BioLegend, Switzerland	1:20
**Secondary antibodies**				
Donkey anti-mouse IgG H&L AF488		ab150105	Abcam, UK	1:400
Donkey anti-rabbit IgG H&L AF488		ab150073	Abcam, UK	1:200
Donkey anti-mouse IgG H&L AF568		ab175472	Abcam, UK	1:400
Anti-rabbit IgG H&L AF568		ab175470	Abcam, UK	1:200
Anti-mouse IgG H&L AF647		ab150107	Abcam, UK	1:200

**Table 3:** Primers used in qRT-PCR analysis.

Gene Name	Forward	Reverse
CCL21	CCATCCCAGCTATCCTGTTCTT	TTCTGTGGGGATGGTGTCTTG
CCL27	CAGACCCTACAGCAGCATTCC	CACGAAAGCCTGGAGGTGAC
CD62P	CCCGAGTCCTTAAGGTTTCCAT	GGAAACAGGGTTGGTCCAGA
CD54	CAGTGACCATCTACAGCTTTCC	CATTCAGCGTCACCTTGGCT
VCAM1	GGAAATGACCTTCATCCCTACCA	ATCTCTGGGGGCAACATTGA
ICAM2	GATTTTGGCAGTGTCGAGGTCT	GGAGCCTGAGGTGTTTCACTTT
F11R	CGAGGCCACTTTGACAGAACA	CCTTCACTTCGGGCACTAGG
TGFBeta1	TGAACCGGCCTTTCCTGCTTCTCATG	GCGGAAGTCAATGTACAGCTGCCGC
CD200/OX2	CCTAAGAATCAGGTGGGGAAGGA	GACGAGAAGAATTACCAGGGAAACA
CD157/BST1	CGCACACTTGCGGGACATC	AGTTCTTGTTCCGCTGCTCG
Podoplanin	AAGAGCTGAAGGGTTACGCC	CACGGGTCATCTTCTCCCAC
Prox1	AAGCAAATGACTTTGAGGTTCC	CAGCTTGCAGATGACCTTGT
GAPDH	AGTCAGCCGCATCTTCTTTT	CCAATACGACCAAATCCGTTG

## Data Availability

The data that support the findings of this study are available on request from the corresponding author.

## Supplementary Materials

The following supporting information can be downloaded at: https://www.mdpi.com/article/10.3390/cells11061055/s1, Figure S1: Evaluation of the expression of blood and lymphatic markers in normal human juvenile/adult (j/a) and fetal (f) skin; Figure S2: Gating strategy involved in the flow cytometry analysis of HDMEC; Figure S3: Gating strategy using isotype controls; Figure S4: Expression of CD200R on human blood immune cells; Figure S5: Expression of CD200R in rat lymph nodes; Figure S6: Expression of CD31 and CD200 in HUVEC; Figure S7: Expression of CD200 in normal a/j human skin. Figure S8: Expression of CD200 in normal a/j human skin; Figure S9: Expression of CD200R in rat lymph nodes.
