# Supplementary material for: The Role of CD200–CD200 Receptor in Human Blood and Lymphatic Endothelial Cells in the Regulation of Skin Tissue Inflammation
Source: Cells. 2022 Mar 21;11(6):1055. doi: 10.3390/cells11061055 (PMC8947338; doi:10.3390/cells11061055)
Supplement: Supplementary file 1 [file cells-11-01055-s001.zip › cells-1562001-supplementary.pdf]

Supplementary Figures

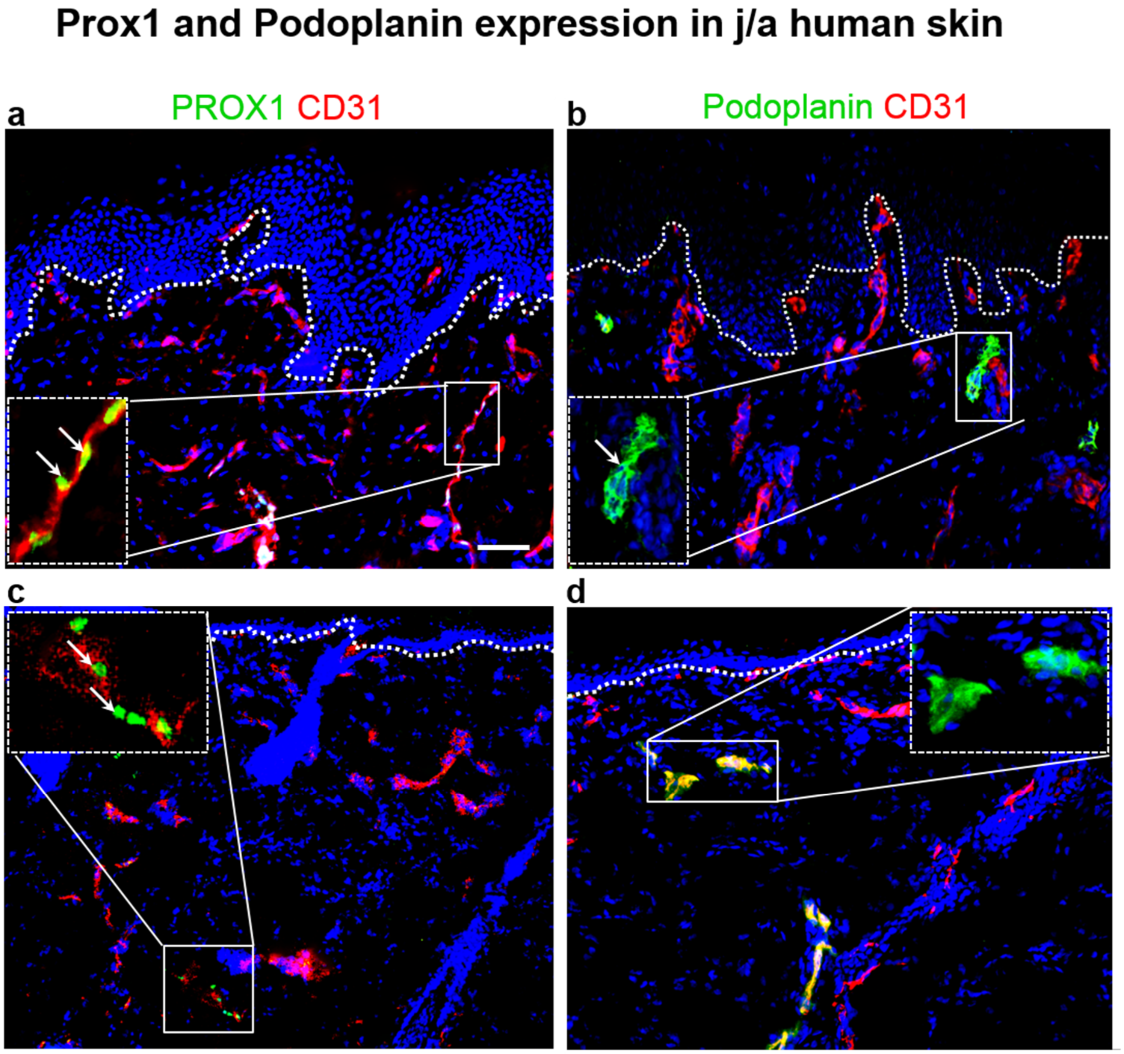

Supplementary Figure S1. Evaluation of the expression of blood and lymphatic markers in normal human juvenile/adult (j/a) and fetal (f) skin. a-d Normal human j/a skin (a, b) and f skin (c, d) were immunostained against CD31 (red) as well as PROX1 (green, a, c) and Podoplanin (green, b, d) both delineating lymphatic capillaries. Insets represent the higher magnification of lymphatic vessels double positive for CD31 and Prox1 or CD31 and Podoplanin. Dotted lines represent the dermo-epidermal junction. Cell nuclei are stained with Hoechst (*blue*). Scale bars: 50 µm.

Gating strategy

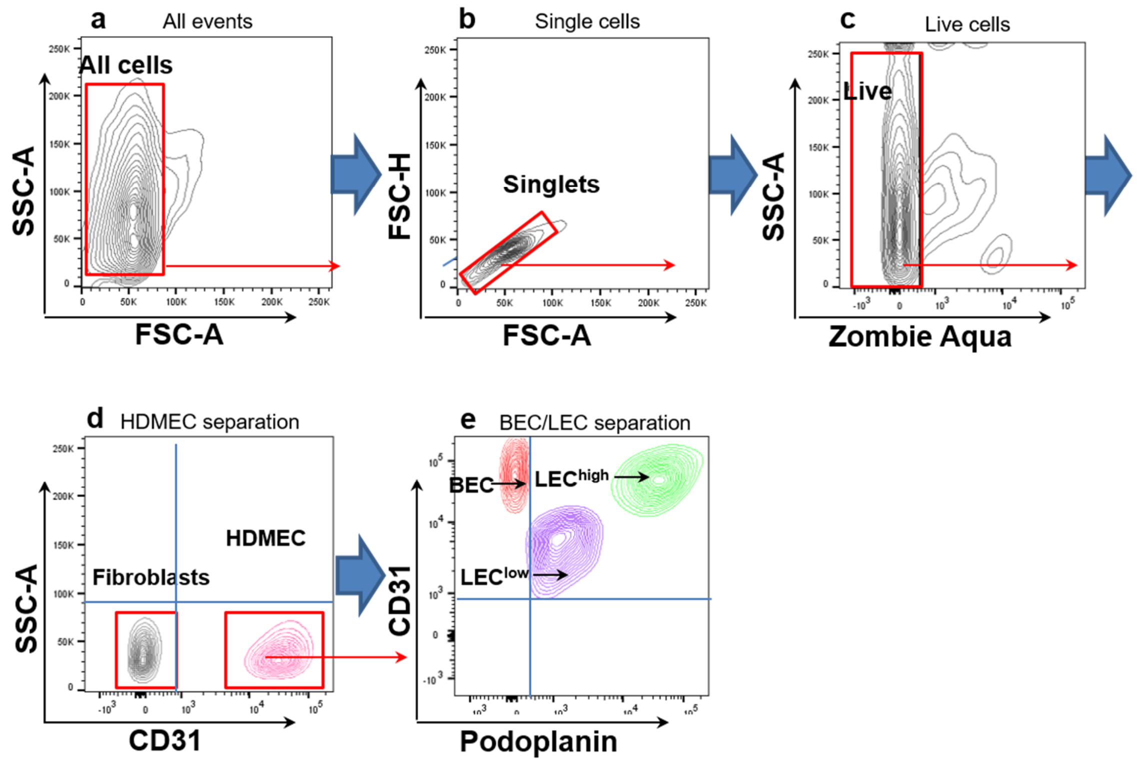

Supplementary Figure S2. Gating strategy involved in the flowcytometry analysis of HDMEC a-c Hierarchical steps during gating strategy involved the exclusion of dead cells using Zombie Aqua staining (a), separation of $CD31^+$ endothelial cells (b) as well as the discrimination between blood and lymphatic endothelial cells based on the Podoplanin expression (c) (BEC and LEC, respectively).

Gating strategy for isotype controls

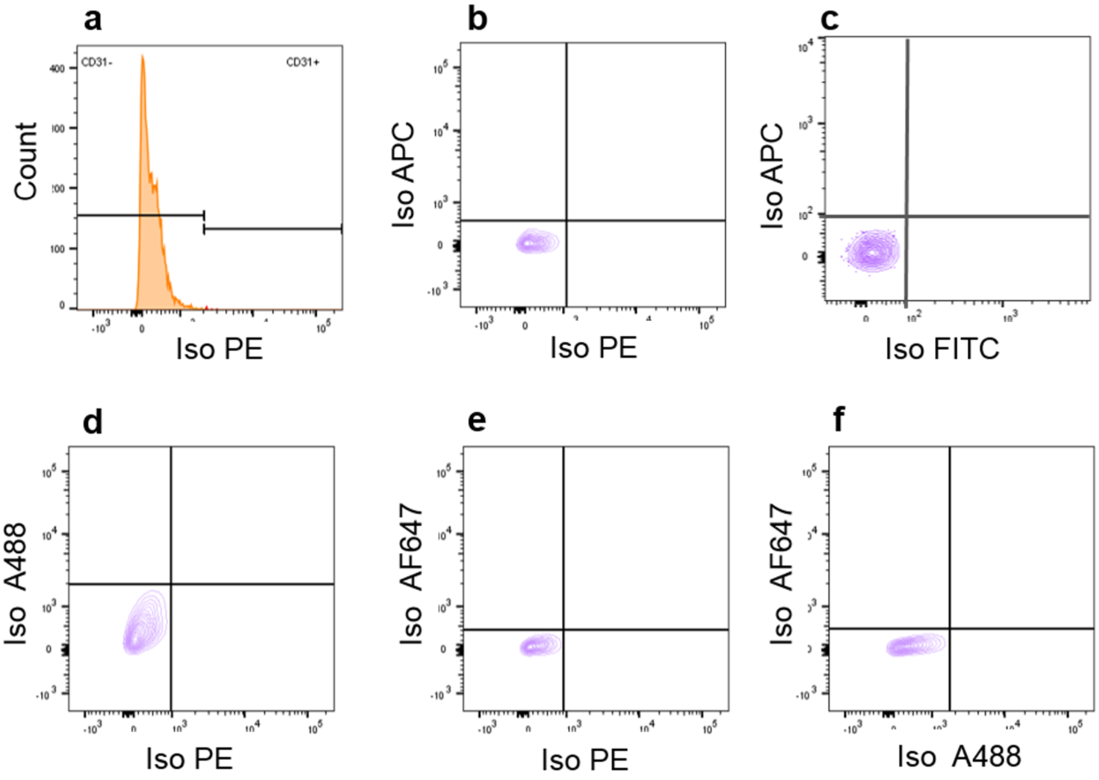

Supplementary Figure S3. Gating strategy for isotype controls. (**a-f) a** Gates based on isotype controls were set such as to include all non-stained cells as a negative population. **b-f** Isotype controls for all fluorophores are shown in a pair-wise manner. The following representative examples for the isotype gating strategy are shown: isotype-matched PE acting as control for CD31, isotype-matched APC acting as control for CD200R, isotype-matched FITC acting as control CD3, isotype-matched AF488 as control for CD200.

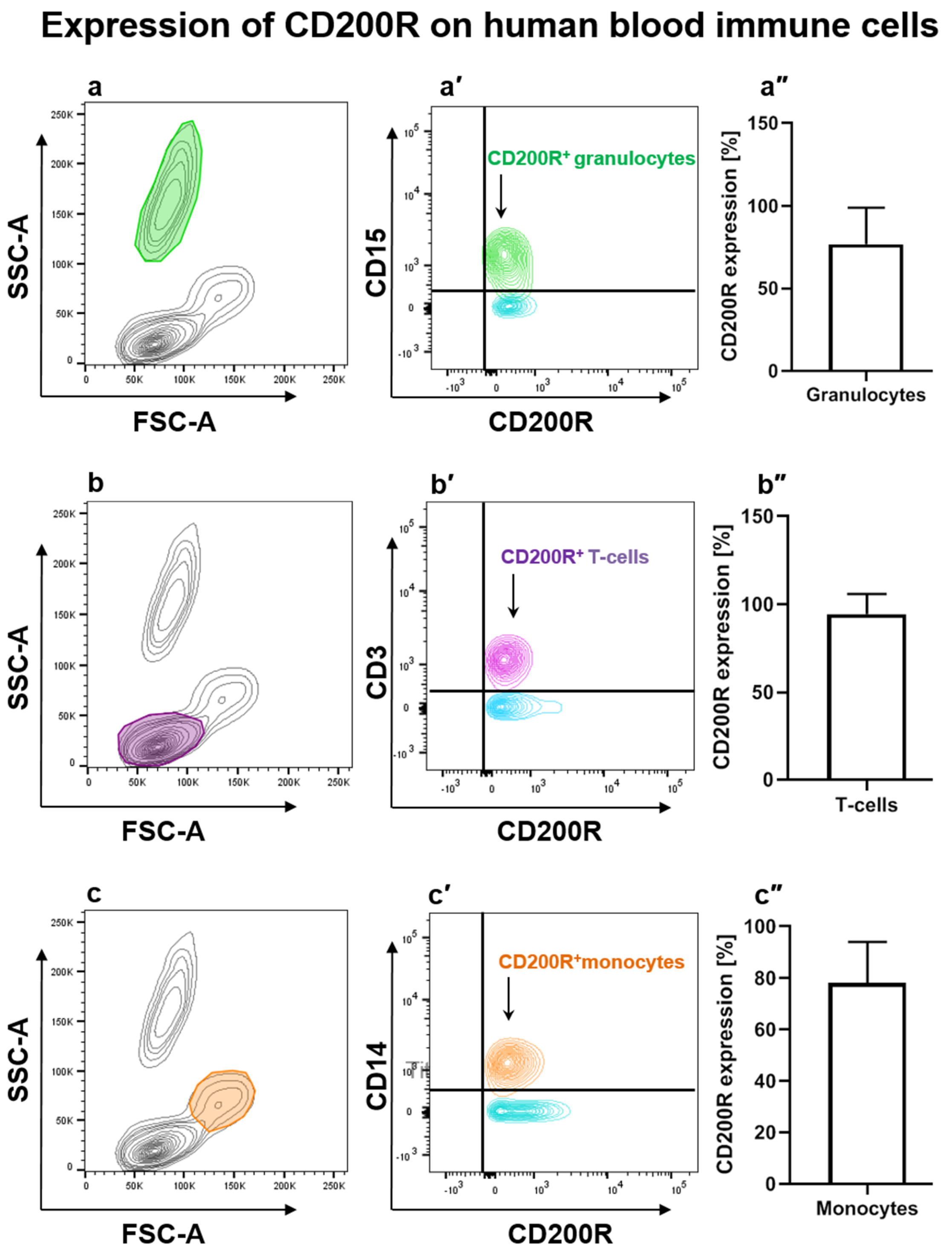

Supplementary Figure S4. Expression of CD200R on human blood immune cells. **a-a''** After the lysis of red blood cells, granulocytes were gated on the forward vs. side scattered plot (a, green). Flow cytometry analysis revealed that 70%±15.7 of all granulocytes exhibit CD200R expression. CD200R^{+}/CD15^{-} cells are shown in cyan. **b-b''** T-cells identified in the forward vs. side scattered plot (b, violet) were further analyzed with respect to the CD200R expression 95%±22.5. Please note that almost all T cells are positive for CD200R. CD200R^{+}/CD3^{-} cells are shown in cyan. **c-c''** The majority of monocytes (yellow, c) showed the expression of CD200R (75%±19.2). CD200R^{+}/CD14^{-} cells are shown in cyan.

Expression of CD31 and CD200 in HUVEC

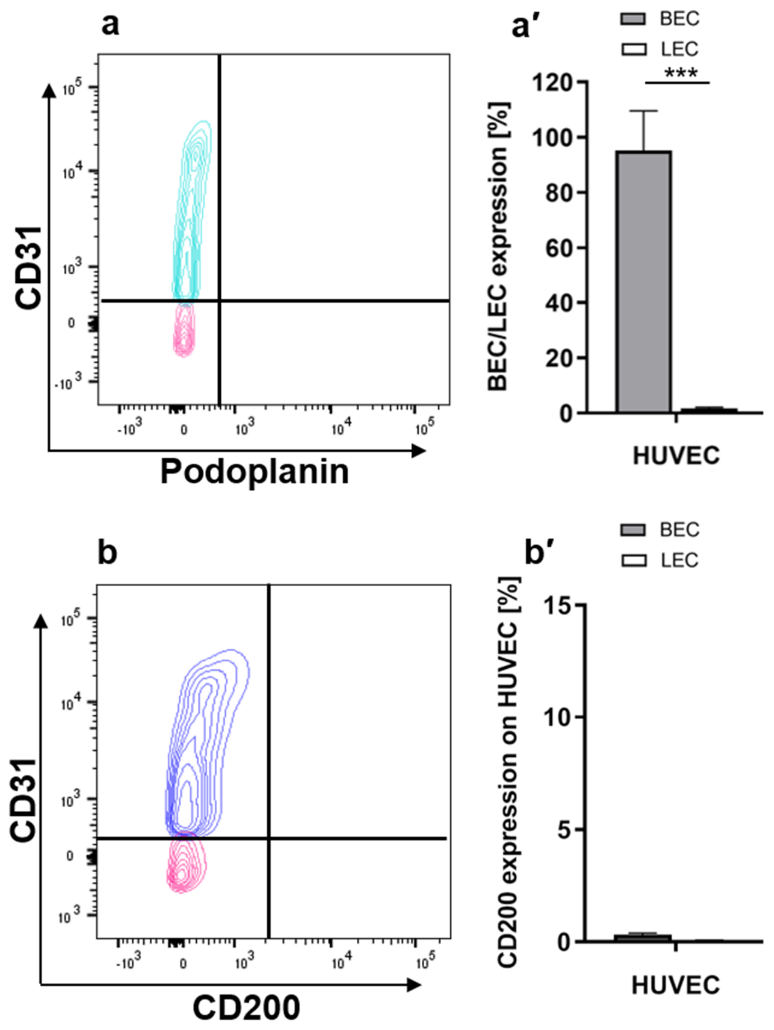

Supplementary Figure S5. Expression of CD31 and CD200 in HUVEC (a-b') Flow cytometry analysis revealed that HUVEC are almost entirely positive for CD31 (a, a'), however, they are negative for Podoplanin (a, a') and CD200 (b, b').

Involvement of CD200-CD200R interaction in the adhesion of human blood T cells to HDMEC

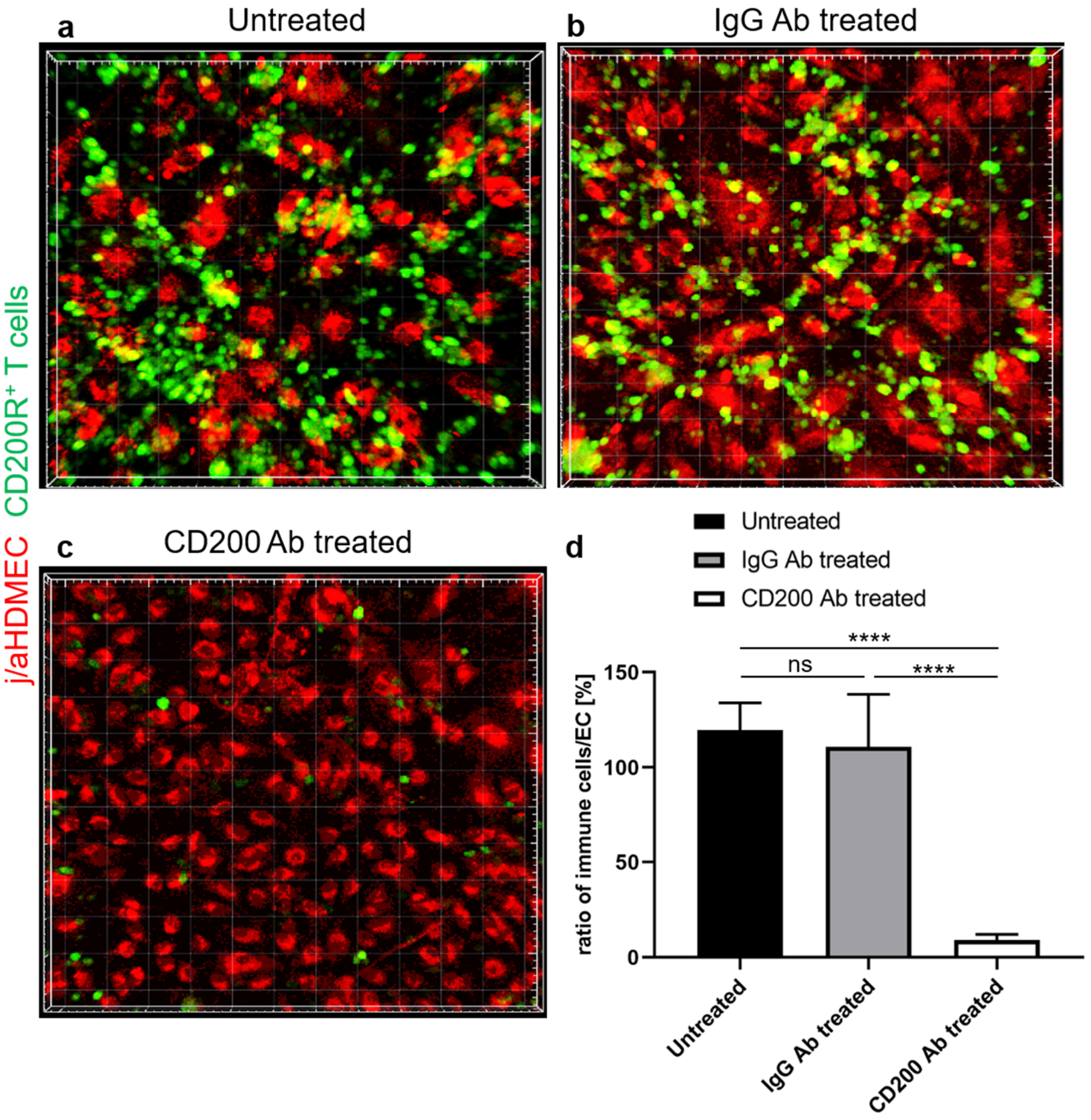

Supplementary Figure S6. Involvement of CD200-CD200R interaction in the adhesion of human blood T cells to HDMEC(a-c) Representative confocal images showing CD200R^{+} T cell binding to adherent j/aHDMEC for untreated (a), IgG antibody treated (b) or CD200 blocking antibody treated (c) conditions. (d) Quantification of adherent cells reveals that the ratio of prelabeled blood T cells/EC is the highest in untreated conditions (119.7%±14.2), with slightly lower values observed in the IgG antibody treated conditions (110.8%±27.6). CD200 blocking antibody treatment significantly decreased the number of binding T cells to 9.4%±3.4 ($p<0.0001$) relative to both, untreated and IgG antibody treated conditions. Asterisks denote significance as follows (unpaired t test): $*p < 0.05$; $**p < 0.01$; $***p < 0.001$; and $****p < 0.0001$. Results are representative of 3 different HDMEC donors, shown are means ± SD.

Control of CD200 blocking efficiency *in vitro*

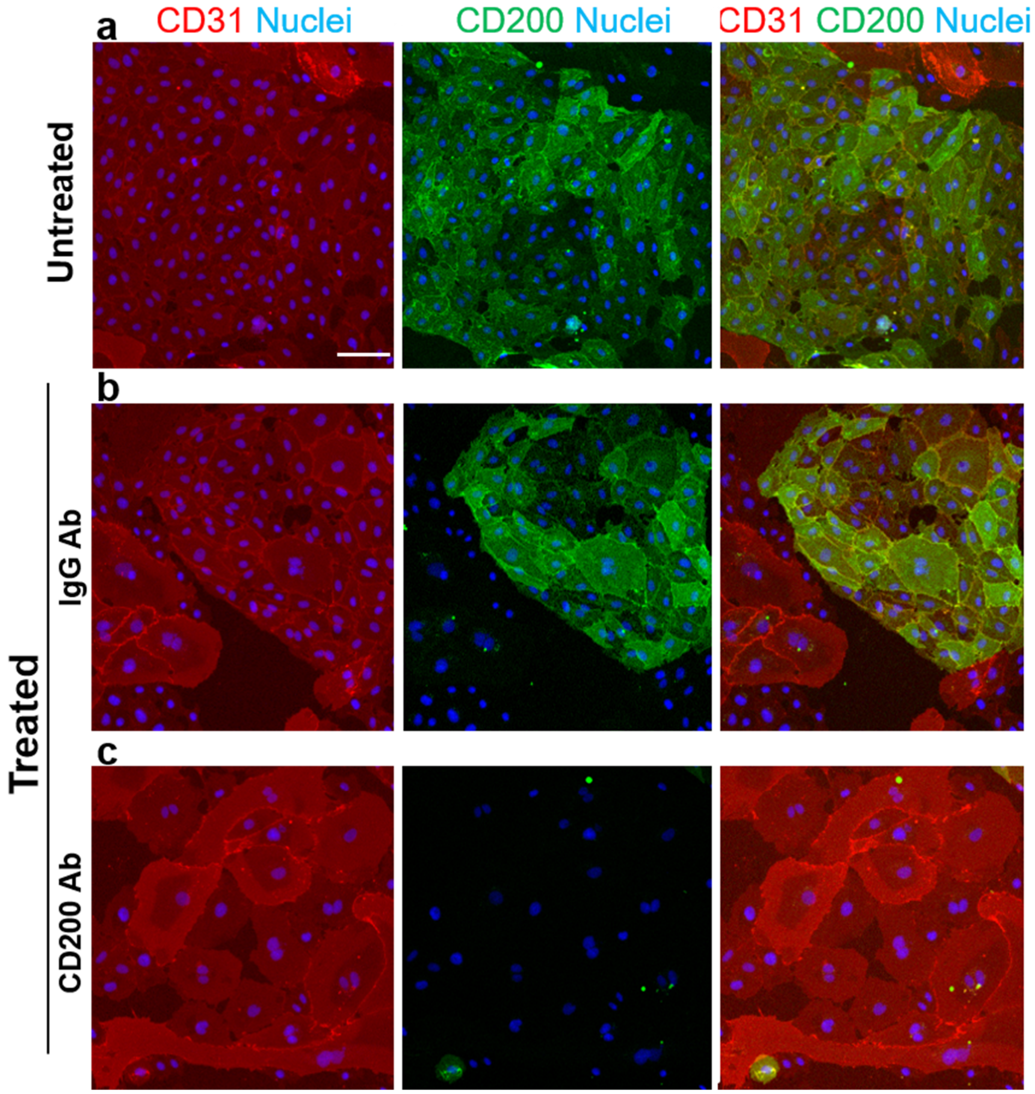

Supplementary Figure S7. Controlof CD200 blocking efficiency in vitro. (a-c) Untreated(a), IgG antibody treated (b) and CD200 blocking antibody treated (c) adherent HDMEC were fixed and then stained with conjugated CD200-AF647. The stainings confirm that CD200 blocking antibody treatment effectively neutralize CD200 epitopes.

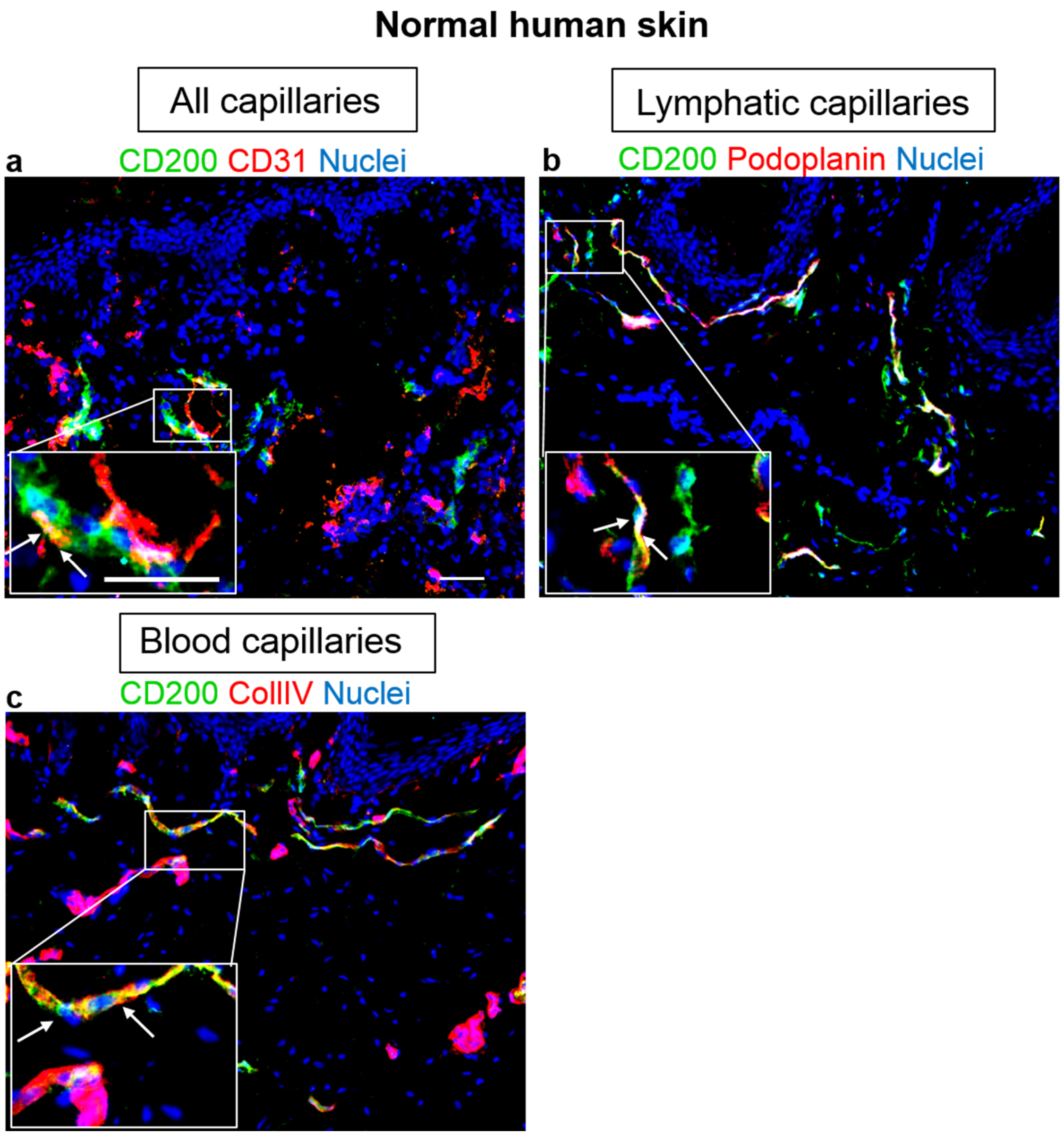

Supplementary Figure S8. Expression of CD200 in normal a/j human skin. a-c Immunofluorescence co-staining of j/a human skin against huCD200 (green) and huCD31 (red) (a) or huPodoplanin (red) (b) or huCollagen IV (red) (c). White arrows in magnification insets indicate double-positive microvascular structures. **a** Immunofluorescence co-staining of j/a skin against huCD200 and huCD31 confirms the expression of CD200 on distinct capillaries. **b** huPodoplanin delineates specifically the human lymphatic capillaries, which are in majority positive for CD marker. **c** huCollagen IV depicts the human blood endothelium, which is only scarcely positive for CD200. Cell nuclei are stained with Hoechst (*blue*). Scale bars: 50 μm.

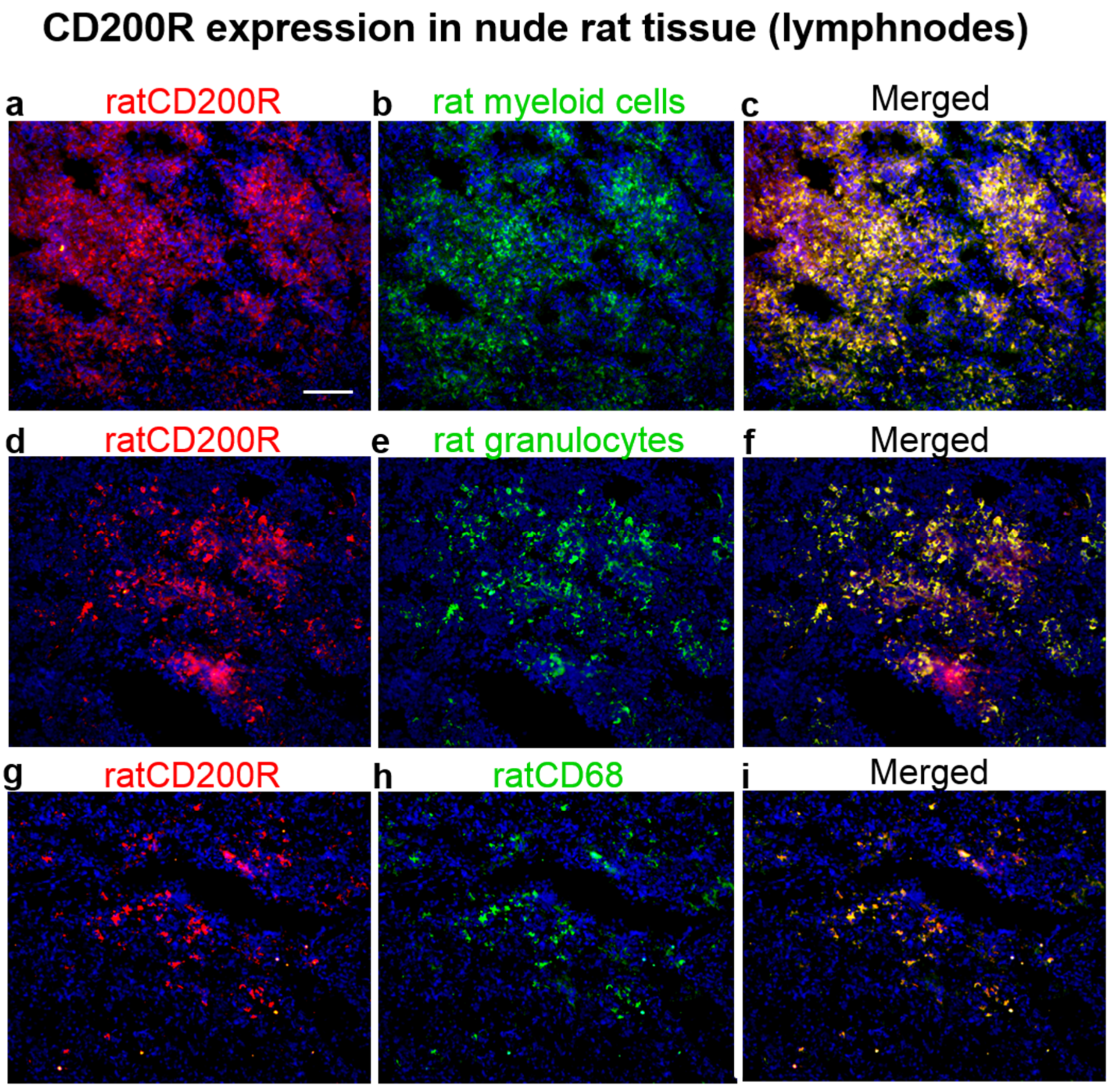

Supplementary Figure S9. Expression of CD200R in rat lymph nodes. a-i Nude rat tissue (lymph nodes) was double stained for CD200R (red) and other immune cell markers, including rat myeloid cells (green, a-c), rat HIS48-granulocytes (green, d-f) as well as rat monocytes/macrophages (CD68) (green, g-i). Please note that rat CD200R-expressing cells are at the same time positive for myeloid, granulocytes, and monocyte/macrophage markers. Cell nuclei are stained with Hoechst (*blue*). Scale bars: 100 µm.
